# Generation of Human Endometrial Assembloids with a Luminal Epithelium using Air–Liquid Interface Culture Methods

**DOI:** 10.1002/advs.202301868

**Published:** 2023-08-27

**Authors:** Jiwen Tian, Jie Yang, Tingwei Chen, Yu Yin, Nan Li, Yunxiu Li, Xingyu Luo, E Dong, Haoyang Tan, Yanping Ma, Tianqing Li

**Affiliations:** ^1^ State Key Laboratory of Primate Biomedical Research Institute of Primate Translational Medicine Kunming University of Science and Technology Kunming Yunnan 650032 China; ^2^ Medical School Kunming University of Science and Technology Kunming Yunnan 650032 China; ^3^ Department of Reproductive Medicine The First People's Hospital of Yunnan Province Kunming Yunnan 650021 China; ^4^ Yunnan Key Laboratory of Primate Biomedical Research Kunming Yunnan 650500 China

**Keywords:** air–liquid interface, endometrial assembloids, luminal epithelium, organoid

## Abstract

The endometrial lining of the uterus is essential for women's reproductive health and consists of several different types of epithelial and stromal cells. Although models such as gland‐like structures (GLSs) and endometrial assembloids (EnAos) are successfully established, they lack an intact luminal epithelium, which makes it difficult to recapitulate endometrial receptivity. Here, a novel EnAo model (ALI‐EnAo) is developed by combining endometrial epithelial cells (EnECs) and stromal cells (EnSCs) and using an improved matrix and air–liquid interface (ALI) culture method. ALI‐EnAos exhibit intact EnSCs and glandular and luminal epithelia, which recapitulates human endometrium anatomy, cell composition, hormone‐induced menstrual cycle changes, gene expression profiles, and dynamic ciliogenesis. The model suggests that EnSCs, together with the extracellular matrix and ALI culture conditions, contribute to EnAo phenotypes and characteristics reflective of the endometrial menstrual cycle. This enables to transcriptionally define endometrial cell subpopulations. It anticipates that ALI‐EnAos will facilitate studies on embryo implantation, and endometrial growth, differentiation, and disease.

## Graphical abstract

By combining endometrial epithelial cells and stromal cells, and using an improved matrix and air–liquid interface (ALI) culture method, a novel ALI‐EnAo (endometrial assembloids) model is developed, which recapitulates the human endometrium in anatomy, cell composition, hormone‐induced menstrual cycle changes, gene expression profiles, and dynamic ciliogenesis. ALI‐EnAos will facilitate studies on embryo implantation and on endometrial growth, differentiation, and disease.

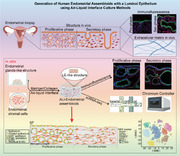

## 1 Introduction

The uterus is an essential organ for human reproduction; its inner lining, the endometrium, is a highly dynamic tissue that undergoes cyclical phases of growth (the proliferative phase, Pro‐phase), differentiation (the secretory phase, Sec‐phase), degeneration (the menstrual phase), and regeneration in response to ovarian hormone changes.^[^ ^1^ ^]^ Abnormalities during endometrial remodeling and regeneration can cause infertility, recurrent pregnancy loss, endometrial tumors, inflammation, a thin endometrium, and endometriosis. Endometrial dysfunction impacts many women of childbearing age. Some defects impair endometrial receptivity during the secretory phase after ovulation, which can lead to infertility.^[^ ^2^ ^]^ However, the regulation of the implantation window^[^ ^3^ ^]^ and the mechanisms underlying implantation are still unclear. Furthermore, results obtained from animal models cannot be easily translated to the human context due to differences in hormonal regulation and the complexities of the human maternal‐fetal interface.^[^ ^2^, ^4^ ^]^ The lack of a reliable and representative model is the most significant hurdle to understanding human endometrial function.

The rapid development of organoid culture technology, which has recently led to advances in the understanding of intestinal and retinal organoid generation, presents a promising approach to study endometrial biology.^[^ ^5^ ^]^ Indeed, gland‐like structures (GLSs) from human and mouse endometrium can be cultured for a long time and recapitulate several characteristics of uterine glands in vivo.^[^ ^6^ ^]^ The endometrium is a multicellular tissue comprising a monolayer of columnar epithelial cells that form the endometrial surface, known as the luminal epithelium (LE), and a structure known as the tubular glandular epithelium (GE) located beneath this. In addition to endometrial epithelial cells (EnECs), the underlying layer also contains many endometrial stromal cells (EnSCs), which are important during implantation and can sometimes contribute to endometrial diseases, such as intrauterine adhesions.^[^ ^3^, ^7^ ^]^ Human EnSCs undergo dramatic morphological and functional differentiation during the window of implantation (WOI).^[^ ^8^ ^]^ They then transform into decidualized EnSCs for acting as embryo quality sensors to select embryos for implantation.^[^ ^9^ ^]^ Endometrial assembloids have previously been generated by combining primary EnSCs or stromal‐like cells from pluripotent stem cells with EnECs^[^ ^10^ ^]^; however, as these assembloids are simply aggregates of two cell types, they lack typical LE‐like structures and endometrial anatomy, which makes it difficult to fully recapitulate the gene expression patterns and functions of the in vivo endometrium, especially during the WOI.

Hormonal regulation is an important aspect of endometrial biology and the menstrual cycle. During the Pro‐phase, estrogen receptor (ESR) and progesterone receptor (PGR) expression gradually increases in both the LE and GE, peaks during ovulation, and then gradually decreases.^[^ ^11^ ^]^ During the Sec‐phase, the progesterone secreted by the corpus luteum antagonizes the actions of estrogen, which transforms the endometrium and makes it more receptive to the adhesion and invasion of an embryo.^[^ ^11^ ^]^ During this period, the expression of temporally‐regulated genes, such as *MUC1*, *LIF*, *ITGB1*, *VEGF*, and *IL‐6*, contributes to the initiation of the WOI.^[^ ^4^, ^12^ ^]^ In addition, during early pregnancy, the LE and GE both contribute to cellular defense and trophoblastic invasion, which helps to maintain normal embryo implantation.^[^ ^13^ ^]^ It is therefore critical to develop a hormone‐responsive endometrial model with complete luminal and glandular structures and a WOI gene signature.

The air–liquid interface (ALI) culture method was initially used to culture epidermal and respiratory tract epithelia.^[^ ^14^ ^]^ With this culture model, the basal surface of the cells is submerged in liquid, while the apical surface is exposed to air, thus mimicking epithelial structures in vivo. Airway epithelial cells grown in this manner form a pseudostratified cell layer with tight connections, cilia, and mucin synthesis abilities, recapitulating the features of their in vivo epithelial counterparts.^[^ ^15^ ^]^ A previous study used the ALI model to establish female reproductive tract polar epithelial cultures to investigate the early embryonic development microenvironment.^[^ ^16^ ^]^ When cultured using the ALI method, murine, porcine, and bovine oviduct epithelial cells form columnar‐shaped epithelia, consisting of secretory and ciliated cells.^[^ ^17^ ^]^ The ALI can therefore support functional epithelial structure formation.

Here, we develop endometrial assembloids that exhibit both LE‐ and GE‐like structures by combining EnECs and EnSCs and using the ALI culture approach with an improved extracellular matrix. Extensive analysis shows that these assembloids accurately recapitulate in vivo endometrial anatomy, cell composition, and gene expression profiles, including WOI genes. Furthermore, our analysis shows that the ALI culture method is critical for achieving the formation of LE structures.

## 2 Results

### 2.1 Endometrial Assembloids Recapitulate In Vivo Endometrium Hormone Responses and Dynamic Ciliation

In this study, we generated gland‐like structures (GLSs) from human endometrium tissue biopsies using a previously reported method.^[^ ^6a^ ^]^ We then confirmed their capacity for long‐term passage, their clonogenic abilities, epithelial characteristics, and hormone responsivity (Figure S1a–i, Supporting Information). The GLSs exhibited similar characteristics to those from previous reports.^[^ ^6^, ^18^ ^]^ We purified the EnSCs by digesting the endometrial biopsies and then propagated the cells in 2D monolayer cultures. We then confirmed their identity^[^ ^19^ ^]^ and decidualization potential (Figure S1j–p, Supporting Information).

Next, we combined the EnSCs and EnECs and examined how the EnSCs impacted the epithelial structure. To optimize the cultures, we assessed different EnEC:EnSC assembly ratios and their effects on GLS formation and growth (Figure S2a,b, Supporting Information). Following serial examinations, we found that an EnEC:EnSC ratio of 1:3 was most favorable for GLS growth (Figure S2b, Supporting Information). The GLSs consisted of columnar structures comprised of tightly arranged epithelial cells, similar to the columnar‐type cells found in the endometrium in vivo, in addition to squamous cell structures composed of squamous epithelium (Figure S2c, Supporting Information).^[^ ^20^ ^]^ The presence of EnSCs affected GLS morphology and significantly promoted the formation of columnar epithelial cells in them (Figure S2c, Supporting Information). Based on these data, we proceeded with a 1:3 assembly ratio for all follow‐up experiments, unless otherwise noted. We named the assembloid of EnSCs and EnECs, “endometrial assembloids” (EnAo).

**Figure 1:**
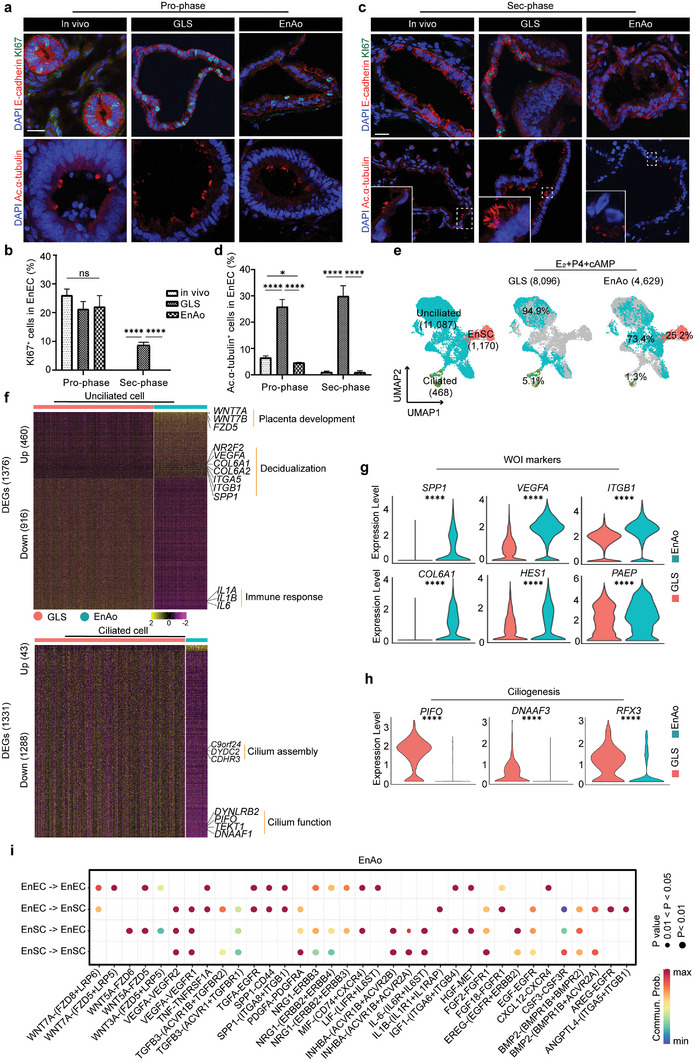
Endometrial stromal cells (EnSCs) contribute to gland‐like epithelium activity. GLS especially indicates gland‐like structures without EnSC incorporation. a) Representative staining of KI67 and acetylated‐α‐tubulin (Ac.α‐tubulin) in the endometrium, gland‐like structure (GLS), and endometrial assembloid (EnAo) at proliferative phase (Pro‐phase). Scale bars: 25 µm. b) Quantification of KI67^+^ cells in EnECs of glandular epithelium across menstrual phases. c) Representative staining of KI67 and Ac.α‐tubulin in the endometrium, GLS and EnAo at secretory phase (Sec‐phase). Scale bars: 25 µm. d) Quantification of Ac.α‐tubulin^+^ ciliated cells in EnECs across menstrual phases. e) UMAP visualization of integrated single‐cell RNA‐Seq from GLSs and EnAos at D9 after E_2_+P4+cAMP treatment (as the protocol of Figure S1d, Supporting Information). f) Heat maps of differentially expressed genes (DEGs) in unciliated cells (top) and ciliated cells (bottom) between GLSs and EnAos. Gene expression levels were normalized. Red represents GLSs and green represents EnAos. g) Violin plots showing expression levels of WOI marker genes in epithelial unciliated cells of GLSs and EnAos. Gene expression levels were normalized. Red represents GLS and green represents EnAo. h) Violin plots showing expression levels of marker genes related ciliogenesis in ciliated cells of GLSs and EnAos. Gene expression levels were normalized. Red represents GLS and green represents EnAo. i) Dot plots of representative ligand‐receptor interactions between EnECs and EnSCs in EnAos. Circle size indicates *P* value and the color means the average expression of the interacting molecules, respectively. b,d) All data were obtained from three different donors. Data are presented as means ± SEMs; Wilcoxon test was used to perform gene expression in scRNA‐seq and two‐sided unpaired Student's *t*‐test was used to perform the statistical analyses of staining; ^*^ *p* ≤ 0.05; ^**^ *p* ≤ 0.01; ^***^ *p* ≤ 0.001; ^****^ *p* ≤ 0.0001; ns, no significance.

We evaluated whether cell behavior within the assembloids was similar to the behaviors of corresponding cells in vivo and explored any GLS variations after adding EnSCs aspects that have not previously been clarified in assembloids.^[^ ^10a^ ^]^ To do this, we examined the responsivity of the cells to hormonal stimulation and analyzed their gene expression profiles. We simulated the in vivo menstrual cycle by treating the EnAos with either estradiol (E_2_) or a combination of E_2_, progesterone (P4), and cyclic adenosine monophosphate (cAMP) for eight and four days,^[^ ^6a^ ^]^ respectively, (Figure S1d, Supporting Information). The E_2_‐treated EnECs in the EnAos displayed similar proliferation abilities to those in the GLSs lacking EnSCs (**Figure** **1**a,b). After being treated with E_2_, P4, and cAMP, EnEC growth in the EnAos was almost completely arrested, consistent with their phenotypes in vivo (Figure 1b,c). In contrast, we observed higher proliferation (≈8%) for the EnECs in the GLSs lacking EnSCs, in accordance with previously reported results (Figure 1b,c).^[^ ^6^, ^21^ ^]^ Our results, therefore, showed that cell proliferative ability in EnAos responded to hormones were more consistent with in vivo observations.

Another hormone‐dependent feature of endometrial cells is ciliation.^[^ ^22^ ^]^ Motile cilia play many roles in epithelial systems. For example, in the human respiratory system and reproductive tracts, they facilitate the flow of fluids by beating in a coordinated manner, are involved in mucus clearance, and enable gamete and embryo transport.^[^ ^18^, ^23^ ^]^ The proportions of ciliated cells in the uterine glands and lumen in vivo vary throughout the menstrual cycle.^[^ ^22^, ^24^ ^]^ Estrogen promotes the growth of ciliated cells, while progesterone, which antagonizes the effects of estrogen, results in de‐ciliation of the cells until the gestational period^[^ ^22b,c,d^ ^]^; this enhances embryo apposition, adhesion, and invasion into the endometrium. Similar to the variation of ciliated cells in vivo, we found that ≈5% of the EnAo cells expressed acetylated‐α‐tubulin^+^—a marker of ciliated cells—during the Pro‐phase. The proportion of ciliated cells then decreased to undetectable levels during the Sec‐phase (Figure 1a,c,d). In contrast, the GLSs lacking EnSCs contained ciliated cells throughout the menstrual cycle, with 25–30% of cells expressing acetylated‐α‐tubulin^+^ (Figure 1a,c,d), consistent with previous results.^[^ ^6^, ^21^, ^25^ ^]^ These results show that EnSCs induce a decrease in the number of ciliated cells and are required for maintaining ciliated cell dynamics throughout the menstrual cycle (Figure 1a,c,d). Moreover, the cilia morphologies in both in vivo and the EnAos transformed to become shorter and flatter during the Sec‐phase (Figure 1a,c); however, these changes were not apparent in the GLSs lacking EnSCs (Figure 1a,c).

To further assess the differences between the decidualized GLSs lacking EnSCs and the decidualized EnAos, we performed scRNA‐seq analyses using the 10x Genomics platform. Cell type annotation using Uniform Manifold Approximation and Projection (UMAP) analysis identified three main groups: ciliated epithelium, unciliated epithelium, and stromal populations, based on typical cell markers (Figure S2d,e, Supporting Information). Integrative analyses revealed significant differences between the gene expression and cell subpopulations within the unciliated epithelial cells (UECs) in the GLSs lacking EnSCs and the EnAos, particularly regarding genes related to the inflammatory response and extracellular matrix (Figure 1e; Figure S2f, Supporting Information). In contrast to the GLSs lacking EnSCs, the UECs in EnAos showed 460 significantly upregulated genes and 916 downregulated genes (Figure 1f; Figure S2g, Supporting Information). The upregulated genes in the EnAo‐UECs were associated with positive regulation of cell adhesion, epithelial migration, vasculogenesis, and decidualization, which are all functional endometrial adaptations for embryo implantation during the Sec‐phase (Figure S2h, Supporting Information).^[^ ^4c^ ^]^ Notably, some genes important for endometrial receptivity were also upregulated, including *SPP1*, *ITGB1*, *HES1*, and *VEGFA* (Figure 1g).^[^ ^1^, ^12^ ^]^ The downregulated genes were related to inflammatory responses, consistent with the sustained downregulation of numerous chemokines and other inflammatory modulators during maternal‐fetal immune tolerance of decidualization (Figure S2h, Supporting Information).^[^ ^4^, ^26^ ^]^ These findings show that the gene expression patterns in the EnAos are reflective of implantation in vivo.

Finally, we examined the characteristics of the ciliated cells. The differentially expressed genes (DEGs) in the ciliated cells of EnAos compared with the GLSs lacking EnSCs included 1288 down‐regulated genes, associated with cilium organization, microtubule‐based movement, and cilium assembly, including *DNAAF3*, *RFX3*, and *PIFO* (Figure 1f,h; Figure S2g,i, Supporting Information).^[^ ^23^ ^]^ We then used Cell Chat to explore putative interactions between subpopulations in the EnAos. Ligand‐receptor interactions within several significantly enriched signal pathways, including WNT, EGF, and IGF, centered around the EnSC‐EnEC and EnEC‐EnSC (Figure 1i), which shows the interactions between the EnECs and EnSCs. These results indicate that EnSCs are crucial to maintaining dynamic hormone‐responsive ciliogenesis throughout the menstrual cycle, and suggest a more accurate representation of in vivo endometrial epithelium dynamics. Additionally, these results indicate that incorporating EnSCs improves the phenotypes and gene expression patterns in EnECs.

### 2.2 A Combination of Matrigel and Collagen Facilitates Physiological EnAo Formation

**Figure 2:**
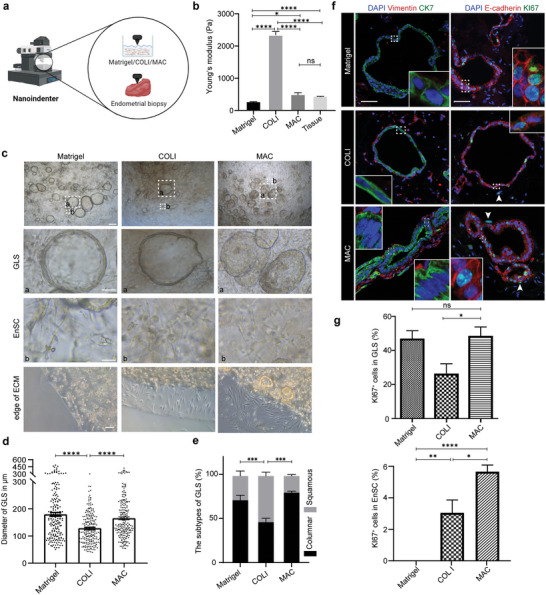
A combination of Matrigel and collagen facilitates physiological EnAo formation. a) Schematic diagram for testing Young's modulus (stiffness) of different culture extracellular matrices (ECMs) containing cells and endometrial biopsies. b) Young's modulus of different ECMs containing cells and endometrium tissue from Pro‐phase. (*N* = 3 donors). c) Representative contrast‐phase images of EnAos growing in different ECMs. Scale bars: 250 µm (low magnification); 100 µm (GLS); 50 µm (EnSC); 100 µm (edge of ECM). d) Quantification of the diameter in µm of GLS growing in different ECMs after hormone treatment on Day 4. At least >180 GLSs were quantified each experiment. Repeated experiments from three donors were assessed, yielding similar results. e) Quantification of columnar‐ and squamous‐Type GLS in EnAos growing in different ECMs. At least >100 GLSs were quantified each experiment. f) Representative double staining of Vimentin/CK7 (left) and KI67/E‐cadherin (right) in EnAos grown in different ECMs. Scale bars: 25 µm. Arrows, KI67^+^ EnSCs. g) Quantification of KI67^+^ cells in GLSs and EnSCs grown in different ECMs. e,g) All data were obtained based on three different donor cells; b,d,e,g), data are presented as means ± SEMs; Chi‐square test was used to analyze percentage of GLS subtypes and two‐sided unpaired Student's *t*‐test was used to perform the statistical analyses of staining and stiffness; ^*^ *p* ≤ 0.05; ^**^ *p* ≤ 0.01; ^***^ *p* ≤ 0.001; ^****^ *p* ≤ 0.0001; ns, no significance.

Physical stiffness in the cellular microenvironment can modulate cell behavior and differentiation.^[^ ^27^ ^]^ We, therefore, queried whether EnAo formation could be enhanced by altering the physical properties of the culture microenvironment. The in vivo endometrial extracellular matrix (ECM) consists of laminin and fibronectin. Collagen is the primary component of the ECM in the outermost layer of the spiral artery and perivascular regions and acts to maintain endometrial stiffness.^[^ ^27^, ^28^ ^]^ Matrigel is a mixture of ≈60% laminin, 30% collagen IV, and 8% entactin, along with other growth factors^[^ ^29^ ^]^ used for culturing GLSs.^[^ ^6^, ^30^ ^]^ To further optimize the ECM for culturing EnAos, we compared three different ECM conditions: Matrigel, Collagen I (COLI), and a mixture of Matrigel and COLI (MAC). We then tested their stiffness and examined the corresponding phenotypes of the EnAos (**Figure** **2**a) (see Experimental Section). As cell growth can lead to measurable changes in the mechanical properties of the matrix,^[^ ^27^ ^]^ we tested the stiffness of the cell‐embedded ECMs after five days of culture with four days of E_2_ treatment (see methods). Under these conditions, the stiffness of Matrigel was ≈279.9 ± 16.07 Pa, which was significantly lower than COLI (2340 ± 112.1 Pa) and MAC (498.1 ± 76.83 Pa) (Figure 2b). The Matrigel and COLI were different from the cell‐free Matrigel (by ≈70–338 Pa)^[^ ^28b^ ^]^ and the cell‐free COLI (about kilopascal order of magnitudes), respectively.^[^ ^31^ ^]^ The stiffness of endometrial tissues during the Pro‐phase fluctuates between 334 and 656 Pa (*N* = 3 donors) (Table S1, Supporting Information) (Figure 2b), according to previous reports.^[^ ^28^, ^31^ ^]^ Therefore, the stiffness values for MAC measured in the presence of cell culture were closest to the values in the in vivo endometrium. Notably, the size of the GLSs of EnAo growing in collagen was clearly reduced compared with those growing in Matrigel or MAC (Figure 2c,d). In addition, cells grown in Matrigel and MAC developed columnar‐type GLSs, while cells grown in COLI mainly generated squamous‐type GLSs (Figure 2e,f). Thus, in terms of stiffness, GLS size, and morphology, MAC produced cultures that most closely resembled the endometrium in vivo.

We next examined the effect of the ECM on cell morphology and proliferation. The EnSCs exhibited different morphologies when embedded in different ECMs, showing a fibroblast‐like morphology in the MAC and COLI matrix, and a shrinking or irregular morphology in the Matrigel (Figure 2c). Migration is an important function of EnSCs. Many of the EnSCs migrated away from the edges of the MAC and COLI, but not from the Matrigel (Figure 2c). Quantification showed that the GLSs in Matrigel and MAC exhibited comparable levels of proliferation, which were higher than those in COLI (Figure 2d,g). However, proliferative EnSCs were only observed in COLI (3%) or MAC (5.6%) (Figure 2f,g). Interestingly, most proliferative EnSCs were located near GLSs (Figure 2f), suggesting that the proliferation of EnSCs may be induced by GLS‐paracrine signaling.^[^ ^32^ ^]^ Together, these results showed that MAC is a physiologically‐relevant ECM condition for culturing EnAos.

### 2.3 Air–Liquid Interface Culture Methods Improve EnAo Features

**Figure 3:**
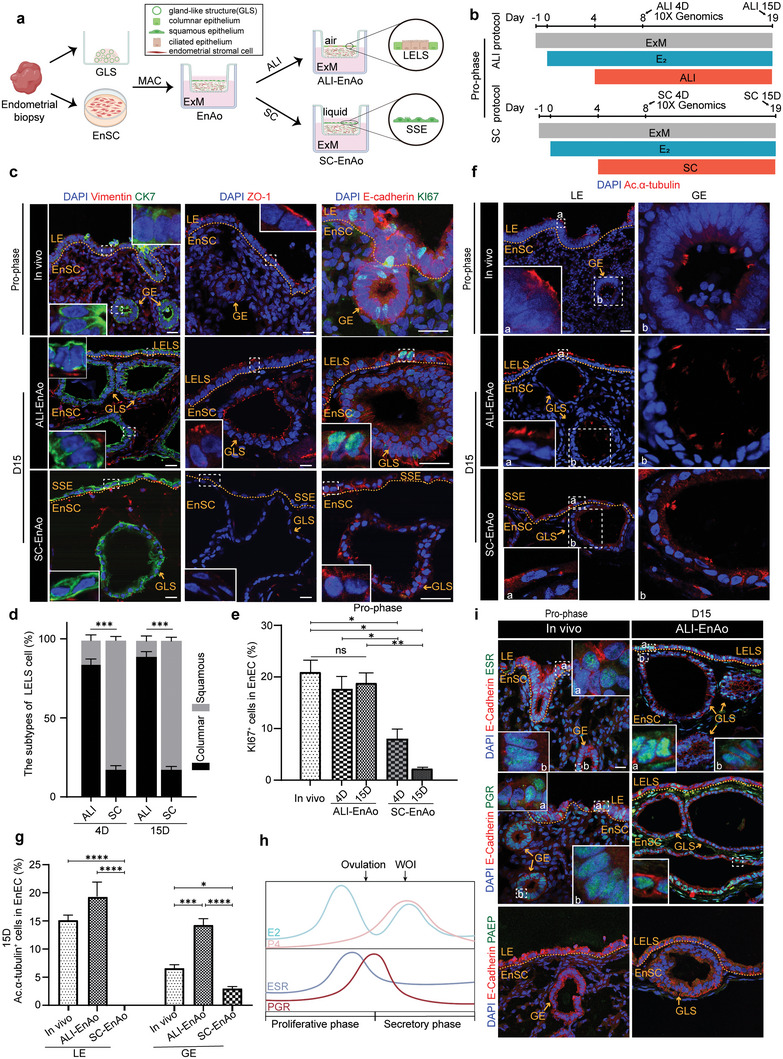
Air–Liquid Interface culture methods improve EnAo features at the Pro‐phase. a) Scheme of the air–liquid interface (ALI) and submerged culture (SC) of EnAos. b) Protocol of ALI‐EnAo and SC‐EnAo to mimic Pro‐phase. The time point at which samples were collected for scRNA‐seq and staining analysis is highlighted with arrows. c) Representative staining of indicated endometrial markers. CK7 for EnECs; Vimentin for EnSCs; ZO1 for cell polarity; KI67 for cell proliferation; and E‐cadherin for epithelium. Scale bars: 25 µm. d) Quantification of columnar and squamous cells in the superficial layer of EnAos cultured in the ALI or SC condition on Day 4 and 15, respectively. At least >150 cells were quantified each experiment. e) Quantification of KI67^+^ EnECs in endometrium in vivo and EnAos cultured in the ALI and SC condition. At least >200 cells were quantified each experiment. f,g) Representative staining f) and quantification g) of ciliated cell marker Ac.α‐tubulin in LE and GE of endometrium, ALI‐EnAos, and SC‐EnAos on D15. Scale bars: 25 µm. h) Dynamic expression changes of estrogen receptor (ESR) and progesterone receptor (PGR) along with E_2_ and P4 change during menstrual cycle. i) Representative staining of indicated markers in endometrium and ALI‐EnAo on D15 at Pro‐phase. Scale bars: 25 µm. d,e,g) All data were obtained based on three different donor cells. Data are presented as means ± SEMs; Chi‐square test was used to analyze percentage of cell subtypes and two‐sided unpaired Student's *t*‐test was used to perform the statistical analyses of staining; ^*^ *p* ≤ 0.05; ^**^ *p* ≤ 0.01; ^***^ *p* ≤ 0.001; ^****^ *p* ≤ 0.0001; ns, no significance. LELS, luminal epithelium‐like structure; GLS, gland‐like structure; SSE, simple squamous epithelium. Endometrium in vivo was obtained from donors during mid‐late Pro‐phase in Figure 3.

We next aimed to refine our culture conditions further to facilitate the formation of functional, hormone‐responsive luminal epithelium‐like structures (LELSs) and GLSs. The LE and superficial GE undergo successive cycles of loss and reconstruction with the menstrual cycle and are important functional units of the endometrium during embryo implantation.^[^ ^33^ ^]^ To enable the EnAos to form functional LELSs and GLSs, we used an air‐liquid surface (ALI) culture system^[^ ^16^ ^]^ for the EnAo cultures to replicate the in vivo context of epithelial structures covered by a mucus layer in the luminal endometrium (**Figure** **3**a,b). We termed this culture system, ALI‐EnAo, to distinguish it from the EnAos that grow in conventional submerged culture (SC‐EnAo).

To explore how hormones impacted morphology, ciliation, and receptor gene expression in the ALI‐ and SC‐grown cells, we examined E2‐treated proliferative ALI‐EnAos and SC‐EnAos on day four (D4) and 15 (D15), representing the early and late stages of the proliferative endometrium, respectively, (Figure 3b). The ALI‐grown cultures substantially promoted GLS expansion, when compared with the SC‐grown cultures (Figure S3a,b, Supporting Information). We then grouped the GLSs into three subtypes, based on morphology and immunostaining results (Figure S3c, Supporting Information). When we compared the Pro‐phase (D15) SC‐EnAo cultures with the ALI‐EnAo cultures, we observed that the ALI‐EnAos strongly promoted the formation of type I GLS, a cystic structure lined by columnar epithelium^[^ ^6a^ ^]^ (Figure 3c; Figure S3a,d, Supporting Information). Columnar LE cells in vivo form tight junctions that exhibit a polarized distribution of zonula occludens‐1 (ZO‐1) (Figure 3c). Similarly, the ALI culture conditions promoted the EnAos to form a LELS with a polarized distribution of ZO‐1 on either D4 or D15 (Figure 3c,d; Figure S3e, Supporting Information). In contrast, the SC conditions resulted in superficial cells arranged in a simple squamous epithelium (SSE) that lacked tight junctions, even after extended culturing, indicating the absence of a typical LE structure (Figure 3c,d; Figure S3e, Supporting Information). Moreover, unlike the weak proliferation of SC‐cultured EnECs, which showed almost no KI67^+^ staining, the ALI‐cultured EnECs with LELSs showed strong proliferation and positive KI67^+^ expression on D4 or D15, similar to epithelial cells in vivo (Figure 3c,e). We also assessed the impact of the ALI cultures on cilium formation. The ALI‐cultured GLSs (ALI‐GLSs) generated ciliated cells, which increased in number from D4 onwards; these results were comparable to GEs in vivo and were markedly higher than results for the SC‐GLSs (Figure 3f,g; Figure S3e,f, Supporting Information). Furthermore, the ALI‐LELS generated ciliated cells on D15, comparable to proliferative LE in vivo, while no ciliated cells were detected with the SC‐SSE (Figure 3f,g; Figure S3e,f, Supporting Information). These results demonstrate that the ALI condition promotes ciliogenesis in EnECs, especially in LELSs. Finally, similar to expression patterns in vivo (Figure 3h), both the Pro‐phase LELSs and GLSs in the E_2_‐treated ALI‐EnAos expressed ESR and PGR, but not progestogen‐associated endometrial protein (PAEP), a secretory‐specific maker (Figure 3i; Figure S3g, Supporting Information). ESR and PGR were also expressed in the GLSs of E_2_‐treated SC‐EnAos, but not in the SC‐SSE (Figure S3h, Supporting Information).

**Figure 4:**
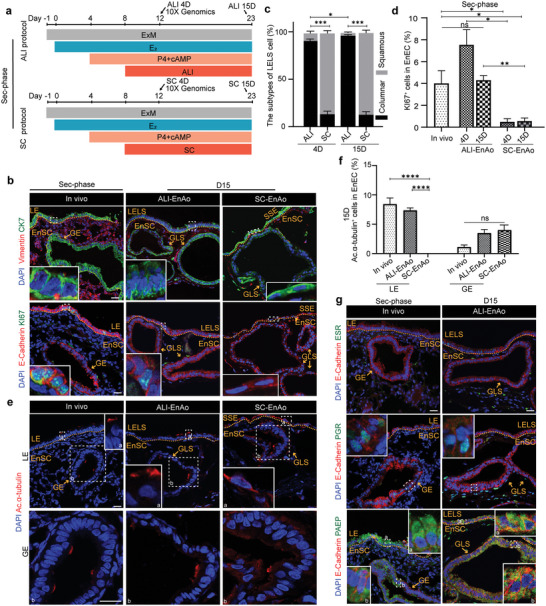
Air–Liquid Interface culture methods improve EnAo features at the Sec‐phase. a) Protocol of ALI‐EnAos and SC‐EnAos to mimic the secretory endometrium in vivo. The time point at which samples were collected for scRNA‐seq and staining analysis are highlighted with arrows. b) Representative staining of endometrial markers in endometrium, ALI‐EnAos, and SC‐EnAos. CK7 for EnECs; and Vimentin for EnSCs; KI67 for cell proliferation; and E‐cadherin for epithelium. Scale bars: 25 µm. c) Quantification of columnar and squamous cells in the superficial layer of ALI‐EnAos and SC‐EnAos on Day 4 and 15, respectively. At least >150 cells were quantified each experiment. d) Quantification of KI67^+^ EnECs in endometrium in vivo, ALI‐EnAos, and SC‐EnAos. At least >200 cells were quantified each experiment. e,f) Representative staining e) and quantification f) of ciliated cell marker Ac.α‐tubulin in LE and GE from endometrium in vivo, ALI‐EnAos and SC‐EnAos on D15. Scale bars: 25 µm. g) Representative staining of E‐cadherin, ESR, PGR, and PAEP in endometrium in vivo and ALI‐EnAo on D15. Scale bars: 25 µm. c,d,f) All data were obtained based on three different donor cells. Data are presented as means ± SEMs; Chi‐square test was used to analyze percentage of cell subtypes and a two‐sided unpaired Student's *t*‐test was used to perform the statistical analyses of staining; ^*^ *p* ≤ 0.05; ^**^ *p* ≤ 0.01; ^***^ *p* ≤ 0.001; ^****^ *p* ≤ 0.0001; ns, no significance. LELS, luminal epithelium‐like structure; GLS, gland‐like structure; SSE, simple squamous epithelium. Endometrium in vivo was obtained from donors during the mid‐late Sec‐phase in Figure 4.

To further test the effects of ALI culture conditions, we treated EnAos with E_2_ for four days, followed by P4 and cAMP for four days; we then cultured the assembloids using either ALI or SC conditions for another four days or 15 days to simulate the secretory endometrium (**Figure** **4**a). We found that the ALI culture method had a significantly positive effect on GLS expansion (Figure S4a,b, Supporting Information). For the SC condition, we observed more abnormal type II GLSs with cell debris and fewer type III GLSs that exhibited folding and tortuosity as representative phenotypes^[^ ^6b^ ^]^ during the Sec‐phase (Figure S4c, Supporting Information). The ALI‐cultured LELSs mainly consisted of columnar or cubic cells, similar to endometrial LE in vivo (Figure 4b; Figure S4d, Supporting Information). Following extended ALI culture, the proportion of columnar epithelial cells increased significantly (Figure 4c). However, almost all of the SC‐cultured epithelial cells on the surfacial layer maintained a squamous arrangement with flattened nuclei and no tight junctions (Figure 4b,c; Figure S4d, Supporting Information), suggesting that the typical LELS layer failed to form. As P4 can antagonize the cell division and ciliogenesis induced by E_2_ treatment,^[^ ^18^, ^25^, ^26^ ^]^ almost all of the GE cells lost their proliferation abilities in vivo, but ≈4–7% of ALI‐LELSs remained positive for KI67, consistent with in vivo phenotypes (Figure 4b,d). In addition, secretory EnECs generated fewer ciliated cells compared to their proliferative counterparts (Figure 3g and Figure 4e,f). The GLS or GE cells from the in vivo, ALI‐EnAos, and SC‐EnAos groups were similar and generated a few ciliated cells with short cilia (Figure 4e,f; Figure S4d–f, Supporting Information). In the ALI‐LELS on D15, we detected ≈7% ciliated cells, which is close to the ≈8% seen with in vivo LEs, but detected no ciliogenesis in the SC‐SSE (Figure 4e,f; Figure S4d,e, Supporting Information). We next examined gene expression patterns after decidualization by treating cultures with P4 and cAMP. We observed a decrease of ESR and PGR and an accumulation of PAEP in the LELSs and GLSs, which further confirmed that decidualized ALI‐EnAos (both on D4 and D15) recapitulate the secretory endometrium (Figure 4g; Figure S4g, Supporting Information). Downregulation of PGR expression in uterine epithelia^[^ ^34^ ^]^ is a common requirement for implantation in mammals as it enables endometrial receptivity (Figure 4g; Figure S4g–i, Supporting Information). Although we observed similar results for the three markers in the GLSs of the Sec‐phase SC‐EnAos, the typical LELS structure was absent (Figure S4h, Supporting Information).

The above statistical quantifications were obtained from three biological replicates; staining for two other donors is shown in Figure S5a,b (Supporting Information), which indicates the reproductivity of ALI‐EnAos.

Together, these results demonstrate that the combination of EnECs and EnSCs grown on a MAC matrix using the ALI culture method generates an endometrial assembloid containing both a LELS and GLS, and this accurately recapitulates the cell composition, anatomy, and menstrual cycle characteristics of the in vivo endometrium.

### 2.4 ALI‐EnAos has a Similar Transcriptome to the In Vivo Endometrium

**Figure 5:**
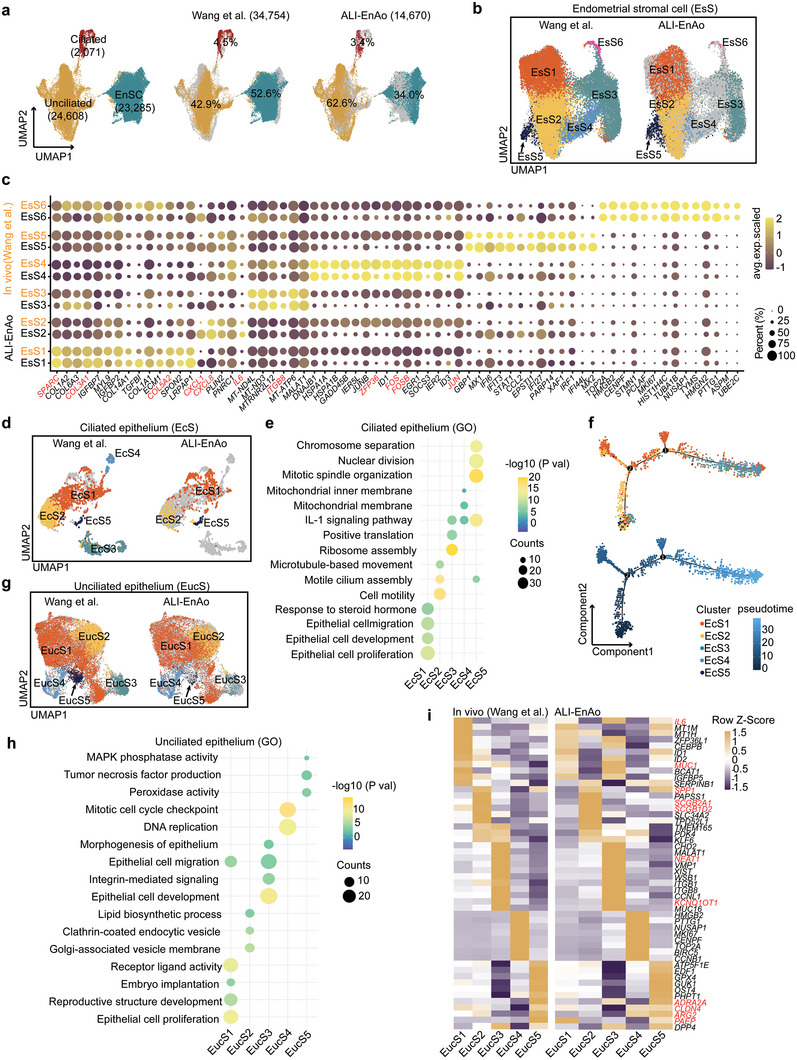
ALI‐EnAos has a similar transcriptome to the in vivo endometrium. a) UMAP analysis of integrated scRNA‐Seq from both Pro‐ and Sec‐phase ALI‐EnAos and human endometrium across menstrual cycle (EnSCs and EnECs from the whole Pro‐ and Sec‐phase).^[^ ^19^ ^]^ b) EnSCs were subclustered into six subtypes, exhibiting high similarity in cell composition between ALI‐EnAos and endometrium. c) Dot plots of some representative genes specific for each subtype in EnSCs of ALI‐EnAos and endometrium in vivo. Dot size indicates proportion of cells expressing the gene in the cluster, and shading indicates the average expression scaled (low to high reflected as light to dark). d) Ciliated cells were subclustered into five subtypes, with high similarity between ALI‐EnAos and endometrium. e) Representative GO terms related to biological processes enriched in different ciliated cell subpopulations. f) Pseudotime showing the differentiation trend of ciliated cell subpopulations. g) Unciliated cells were subclustered into five subtypes, exhibiting high similarity in cell composition between ALI‐EnAos and endometrium. h) Representative GO terms related to biological processes enriched in different unciliated cell subpopulations. i) Composite heatmaps showing relative expression (Z‐scores) of marker genes in unciliated cells subpopulations of ALI‐EnAos and endometrium in vivo.

The results of our analyses on structure and marker genes suggest that ALI‐EnAos recapitulates the in vivo endometrium. To determine if the similarities extended to the molecular level, we conducted a transcriptomic analysis of the ALI‐EnAos during Pro‐phase and Sec‐phase by single‐cell RNA‐seq (scRNA‐Seq). We used ALI‐EnAos treated with E_2_ for eight days to represent the proliferative endometrium (ALI‐EnAos on D4 in Pro‐phase) (Figure 3b). We then used ALI‐EnAos treated with P4 and cAMP for eight days to represent the secretory phase (ALI‐EnAos on D4 in Sec‐phase) (Figure 4a); this was potentially equivalent to the middle secretory endometrium 6–9 days after the LH surge in vivo. We dissociated ALI‐EnAos into single cells and performed scRNA‐seq using the 10x Genomics platform. The transcriptomes of single cells for Pro‐ and Sec‐phase human ALI‐EnAos were generated following quality control and filtration. We also integrated 10x Genomics data from whole proliferative and secretory endometrium in vivo as a reference^[^ ^19^ ^]^ into ALI‐EnAos (Figure S6a, Supporting Information). We then made a comparative analysis between the ALI‐EnAos and in vivo endometrium^[^ ^19^ ^]^ that removed immune cells, endothelial cells, and smooth muscle cells (**Figure** **5**a). Our integrated analyses identified three main cell types based on gene expression patterns (Figure S6b, Supporting Information), all of which were clearly represented in both the ALI‐EnAo and in vivo endometrium (Figure 5a; Figure S6a, Supporting Information).

To better understand the cellular subpopulations in the ALI‐EnAos and the in vivo endometrium, we subclustered the three main cell types and performed further characterizations. The EnSCs were subclustered into six subtypes according to gene marker expression and GO categories; all of these were clearly represented in both the ALI‐EnAo and in vivo endometrium (Figure 5b,c; Figure S6c, Supporting Information). The dominant EnSC subpopulation (EsS1) expressed extracellular matrix and collagen fibril organization marker genes, including *SPARC*, *COL3A1*, and *COL5A2* (Figure 5c; Figure S6c, Supporting Information). The EsS2 subpopulation, which expressed the *CXCL8*, *CXCL1*, and *IL6* genes, was presumed to mainly exert immune regulatory functions (Figure 5c; Figure S6c, Supporting Information), consistent with the immune tolerance of EnSCs and their interactions with immune cells during menstruation, repair, and pregnancy.^[^ ^35^ ^]^ The EsS3 subpopulation expressed the *ITGB8* and *PTGS2* genes and showed enrichment for TGF‐β production and apicolateral plasma membrane‐related gene expression (Figure 5c; Figure S6c, Supporting Information). The genes specific to the EsS4 subpopulation, including *FOS*, *FOSB*, *JUN*, and *ZFP36*, were related to oxidative stress responses, regulation of hemopoiesis, and cellular responses to EGF, suggesting that this subpopulation was highly differentiated and may play a role in tissue repair and regeneration (Figure 5c; Figure S6c, Supporting Information). Notably, the transcriptomic profiles of the EsS5 subpopulation showed enrichment of embryo implantation‐ and cellular senescence‐related genes (Figure S6c, Supporting Information),^[^ ^10a^ ^]^ suggesting that this subpopulation may be involved in embryo implantation. EsS6 was a proliferative subpopulation, that expressed cell cycle genes (Figure 5c; Figure S6c, Supporting Information). Thus, the ALI‐EnAos recapitulated the EnSC subpopulations with a diverse array of functions.

To further define the endometrium cell composition, we also analyzed ciliated cells. These cells were subclustered into five subpopulations (EcS1–5) (Figure 5d). GO analysis showed that the EcS1 subpopulation was involved in epithelial cell development and proliferation; the EcS2 subpopulation in cilium assembly, cell motility, and microtubule‐based movement; and the EcS5 subpopulation represented actively dividing ciliated cells that were only present in Pro‐phase ciliated cells (Figure 5e; Figure S6d, Supporting Information). Two of the clusters (EcS3 and EcS4), which we observed in the human endometrium, were not represented in ALI‐EnAos (Figure 5d). The two missing clusters were associated with protein synthesis and cell stress, respectively (Figure 5e). Pseudotime analysis revealed that these two subpopulations mostly belonged to matured cells (Figure 5f). Their absence in the ALI‐EnAos may reflect the short culture time used with ALI, which resulted in incomplete differentiation. We also found that the genes and unique molecular identifiers (UMIs) in EcS3 and EcS4 were significantly lower than those in other subpopulations, suggesting that these two subpopulations may have been affected by noise from the single‐cell preparation or RNA‐Seq technology (Figure S6e, Supporting Information). Consistent with in vivo ciliated cell trends throughout the menstrual phases, the highest representation of ciliated cells in the ALI‐EnAos treated with P4 and cAMP was in the later time points of the pseudotime analysis (Figure S6f, Supporting Information).

To complete the cellular analysis, we examined epithelial unciliated cells (EucS). Subclustering revealed a high similarity between the EucS of the ALI‐EnAo and its in vivo endometrial counterparts (Figure 5g). The unciliated compartment harbored differentiated epithelial cells (EucS1) involved in epithelial cell proliferation, receptor‐ligand activity, and embryo implantation (Figure 5h), and expressed receptivity‐regulated genes, including *MUC1* and *IL6* (Figure 5i). EucS2 likely represents a subpopulation specialized in secretion, as it specifically expressed two secretoglobin family genes (*SCGB2A1* and *SCGB1D2*),^[^ ^25^ ^]^ and was enriched with Golgi‐associated vesicle membrane and endocytic vesicles (Figure 5h,i).^[^ ^19^ ^]^ Genes in the EucS3 subpopulation were associated with epithelial cell migration, development, morphogenesis, and epithelial‐mesenchymal transition (EMT) (Figure 5h). Notably, the *NEAT1* and *KCNQ1OT1* genes are associated with EMT (Figure 5i).^[^ ^36^ ^]^ The unciliated cells in ALI‐EnAos also included an actively dividing EucS4 subpopulation (Figure 5i). The final subpopulation, EucS5, expressed canonical endometrial receptivity genes (*ADRA2A*, *CLDN4*, *ARG2*, and *PAEP*), which are indicators of the WOI (Figure 5i),^[^ ^12b^ ^]^ and genes related to peroxidase and MAPK phosphatase activity (Figure 5h). The activation of these two key enzymes is associated with the senescence‐associated secretory phenotype (SASP), induced by progesterone. This suggests this subpopulation is involved in physiological tissue remodeling, embryo development, and placenta formation.^[^ ^37^ ^]^ *DPP4* (dipeptidyl peptidase 4), a typical marker of SASP,^[^ ^38^ ^]^ was expressed in the EucS3 and EucS5 subpopulations (Figure 5h), indicating that EucS3 may be involved in implantation.

To summarize, not only did our RNA‐seq analyses show that the ALI‐EnAo exhibited similarity to the in vivo endometrium in terms of cell composition and gene expression, but it also constitutes an atlas of endometrial cell populations and their corresponding transcriptomes.

### 2.5 Identification of Luminal and Glandular Epithelia in ALI‐EnAos

**Figure 6:**
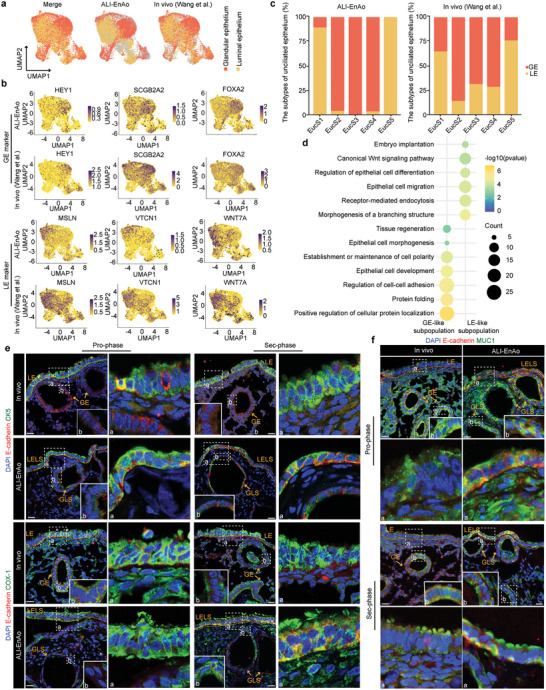
Identification of luminal and glandular epithelium in ALI‐EnAos. a) Annotated unciliated epithelia into luminal and glandular epithelia populations of ALI‐EnAo in the integrative analysis data with in vivo endometrium according to LE and GE specific markers expression. Distribution of LE and GE of in vivo endometrium was shown based on a previous report.^[^ ^19^ ^]^ b) Similar expression pattern of LE (*WNT7A*, *MSLN*, and *VTCN1*) and GE‐specific markers (*HEY1*, *SCGB2A2*, and *FOXA2*) between ALI‐EnAo and in vivo. c) The relationship between EucS1‐EucS5 and LE/GE membership defined in ALI‐EnAo and in vivo, respectively. d) Representative GO terms of genes enriched in LE/GE‐like subpopulation of ALI‐EnAos, respectively. e) Representative staining of indicated luminal epithelium markers for ALI‐EnAos and in vivo endometrium. E‐cadherin for epithelium; CK5 and COX‐1 for luminal epithelium. Scale bars: 25 µm. f) Representative staining of indicated secreted mucus proteins. E‐cadherin for epithelium; MUC1 for mucus protein. Scale bars: 25 µm. LELS, luminal epithelium‐like structure; GLS, gland‐like structure.

In unciliated epithelia in vivo, there is a perpendicular segregation of the luminal and glandular epithelia cells throughout the menstrual cycle.^[^ ^19^ ^]^ To confirm LE and GE presence in the unciliated epithelia of ALI‐EnAos, we integrated data from ALI‐EnAos unciliated epithelia and the in vivo endometrium,^[^ ^19^ ^]^ and then labeled the in vivo LE/GE populations according to previously reported definitions based on their in vivo transcriptome and staining (**Figure** **6**a).^[^ ^19^ ^]^ We then annotated the unciliated epithelia for luminal and glandular epithelia populations in ALI‐EnAos according to gene expression patterns and canonical LE and GE markers (Figure 6a,b; see Experimental Section; Figure S6g, Supporting Information). *WNT7A*, a gene highly expressed in primate^[^ ^39^ ^]^ and human^[^ ^19^, ^25^, ^40^ ^]^ luminal epithelia, was mainly expressed in putative LE subpopulations (Figure 6a,b). Other LE markers, including *VTCN1* ^[^ ^19^ ^]^ and *MSLN*,^[^ ^25^, ^41^ ^]^ were also specially expressed in the putative LE subpopulations (Figure 6a,b). Conversely, *HEY1*,^[^ ^19^, ^42^ ^]^ *SCGB2A2* ^[^ ^25^ ^]^ and *FOXA2* ^[^ ^19^, ^25^ ^]^ were highly expressed in putative GE subpopulations (Figure 6a,b). The similar expression patterns of LE‐ and GE‐specific markers (Figure 6a,b) suggested that the distribution of the LE‐ and GE‐like subpopulations in ALI‐EnAos was consistent with the in vivo endometrium, as defined by previous reports.^[^ ^19^ ^]^ Notably, we observed some obvious differences in LE distribution between the ALI‐EnAos and the in vivo endometrium (Figure 6a,b). This may be because the in vivo samples spanned five different phases of the menstrual cycle (proliferative, early, early‐middle, middle, and late secretory), whereas the EnAos only consisted of two phases. Therefore, we described LE/GE subpopulations as LE‐like/GE‐like subpopulations. Additionally, the LE and GE defined in both the ALI‐EnAos and the in vivo endometrium were slightly mixed, which may be due to differences in the EucS1–5 subpopulations rather than LE and GE. EucS1 and EucS5 of the ALI‐EnAos were mainly located in the LE‐like subpopulation, while EucS2–4 were mainly distributed in the GE‐like subpopulation, similar to their distributions in vivo (Figure 6c).

GO analysis showed that genes enriched in the LE‐like subpopulation in the ALI‐EnAos were highly associated with epithelial cell migration, receptor‐mediated endocytosis, and embryo implantation, as well as LE development and differentiation, including genes associated with morphogenesis of a branching structure, and differentiation regulation (Figure 6d); these results were consistent with LE functions in vivo.^[^ ^19^ ^]^ We also observed positive canonical WNT signaling pathway regulation in the LE‐like subpopulation (Figure 6d).^[^ ^25^ ^]^ The genes enriched in the ALI‐EnAos GE‐like subpopulation were associated with protein synthesis and secretion, which are typical GE functions (Figure 6d).

To further identify LELS, we performed immunostaining with LE markers. Both COX1 and KRT5 were especially distributed in the LELS of either Pro‐ or Sec‐phase ALI‐EnAos (Figure 6e), similar to in vivo LE expression patterns.^[^ ^25^ ^]^ Additionally, MUC1 staining revealed that ALI‐EnAos LE cells could produce mucus (Figure 6f). Collectively, our results demonstrate that ALI‐EnAos form typical LELSs and GLSs, similar to the in vivo endometrial epithelium.

### 2.6 ALI Promotes Physiologically‐Relevant Gene Expression Patterns in EnAos

**Figure 7:**
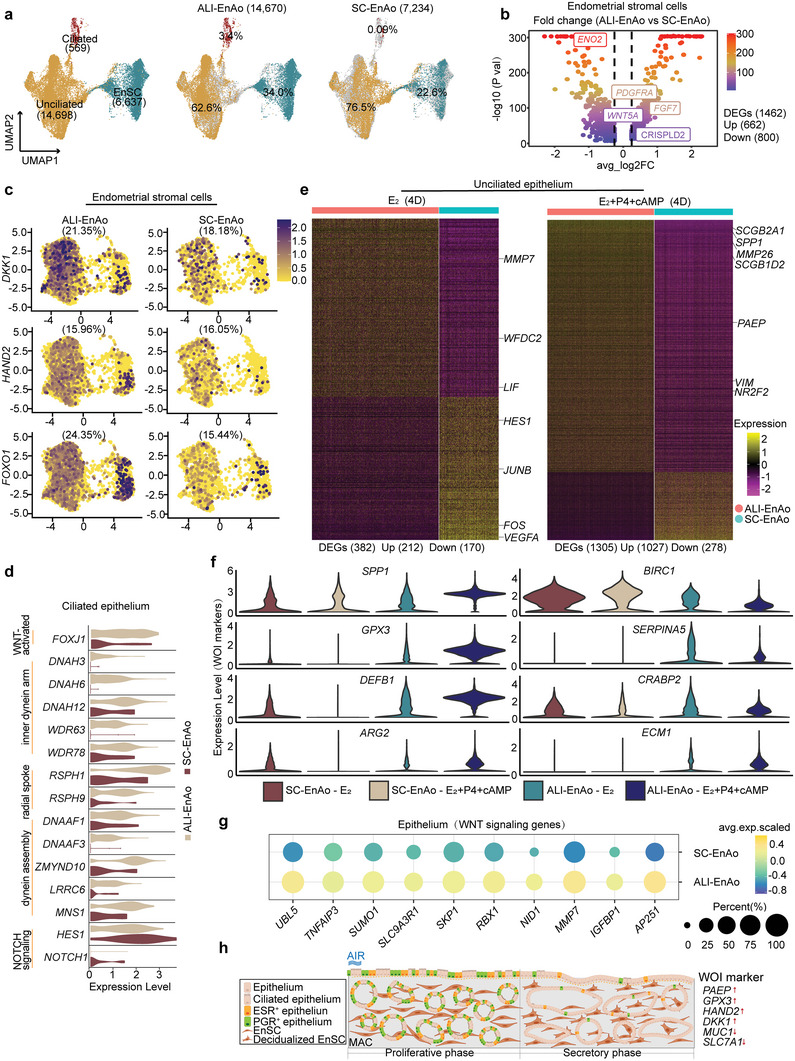
ALI promotes physiologically‐relevant gene expression patterns in EnAos. a) UMAP analysis of integrated scRNA‐Seq data from ALI‐EnAos and SC‐EnAos (both under E_2_ and E_2_+P4+cAMP treatment with ALI 4D). b) Volcano plot of differentially expressed genes (DEGs) in EnSCs between ALI‐EnAos and SC‐EnAos. c) UMAP plots of representative decidualization genes in EnSCs of ALI‐EnAos and SC‐EnAos (The percentage represents the proportion of positively expressed cells). d) Violin plots of ciliogenesis‐related genes in ciliated cells of ALI‐EnAos and SC‐EnAos. e) Heat maps of DEGs in unciliated cells of ALI‐EnAos and SC‐EnAos under E_2_ and E_2_+P4+cAMP treatment to minic different phases. Some representative genes were shown. Gene expression levels were normalized. f) Differential expressions of some representative window of implantation (WOI) genes in ALI‐EnAos and SC‐EnAos under E_2_ (D4) and E_2_+P4+cAMP (D4) treatment to minic Pro‐phase and Sec‐phase. Gene expression levels were normalized. g) Related genes of WNT signaling pathway in epithelium of ALI‐EnAos and SC‐EnAos. h) Schematic diagram of the ALI‐cultured EnAos mimicking proliferative and secretory endometrium.

To illustrate that ALI‐EnAos more closely recapitulate the in vivo endometrium than SC‐EnAos, we compared their single‐cell transcriptomes following E_2_ and E_2_+P4+cAMP treatment. This analysis showed differences in the distribution of three main cell types: EnSCs, unciliated cells, and ciliated cells (**Figure** **7**a; Figure S7a, Supporting Information).

We then identified DEGs in the three cell types between the SC‐EnAos and ALI‐EnAos. In the ALI‐cultured EnSCs, there were 662 significantly upregulated genes and 800 downregulated genes (Figure 7b). The upregulated genes included *FGF7, PDGFRA, CRISPLD2*, and *WNT5A* (Figure 7b),^[^ ^10^, ^19^, ^25^ ^]^ were associated with the non‐canonical WNT signaling pathway, female pregnancy, and response to steroid hormone (Figure S7b, Supporting Information). Prolactin (PRL) and insulin‐like growth factor‐binding protein 1 (IGFBP‐1)^[^ ^8^ ^]^ are important markers of decidualized EnSCs. Two major factors, forkhead box O1 (*FOXO1*) and neural crest derivatives expressed transcript 2 (*HAND2*), are also critical for EnSC decidualization.^[^ ^8^, ^43^ ^]^ Decidualized EnSCs regulate the secretory endothelium by secreting DKK1 to inhibit the WNT pathway.^[^ ^25^ ^]^ Expression of the four genes mentioned above, apart from *HAND2*, was significantly increased (*p* < 0.05) in the ALI‐EnSCs compared with the SC‐EnSCs (Figure 7c; Figure S7c, Supporting Information).

In accordance with the immunostaining results, ciliated cells constituted another key differential subpopulation (Figure 7a). As ciliogenesis is mainly induced by E_2_ and cilia functions are related to motility, we specifically examined genes associated with cilia movement and ciliogenesis during Pro‐phase. Interestingly, compared to SC‐EnAos, genes related to the inner dynein arm, radial spoke, and dynein assembly^[^ ^18^, ^22^, ^44^ ^]^ were specifically expressed (*p* < 0.05) in ALI‐EnAos (Figure 7d), suggesting that the ALI may promote ciliogenesis by regulating structural and regulatory cilia proteins.

The unciliated epithelium also showed significant differences. We observed 839 upregulated genes in the ALI‐cultured unciliated epithelium compared with SC‐cultured cells. These genes were associated with the establishment of tissue polarity, morphogenesis of a polarized epithelium, the WNT signaling pathway, and embryo implantation (Figure S7d,e, Supporting Information). WNT signaling activity is closely related to endometrial LE formation.^[^ ^25^ ^]^ The upregulated genes were associated with these biological processes, including the formation of polar LE in ALI‐EnAos. There were 852 downregulated genes in ALI‐EnAos, mainly associated with glycolytic processes and vasculogenesis (Figure S7d,e, Supporting Information).

An important characteristic of the endometrium is its response to hormonal fluctuations. As gene expression in UECs changes throughout the menstrual phases, we analyzed the DEGs between ALI‐UECs and SC‐UECs following E_2_ and E_2_+P4+cAMP treatment (Figure 7e). In the E_2_‐treated ALI‐UECs, upregulated genes included *WFDC2* and *LIF*, which are mainly associated with LE markers, while the downregulated genes included *FOS* and *JUNB*, which are associated with cell apoptosis, and *VEGFA*, which is related to higher oxygen concentrations (Figure 7e).^[^ ^45^ ^]^ For the E_2_+P4+cAMP‐treated ALI‐UECs, upregulated genes included *SCGB2A*, *SCGB1D2*, and *PAEP*, which encode secretory proteins, and EMT markers, *NR2F2* and *VIM* (Figure 7e).^[^ ^46^ ^]^ This indicates that ALI cultures facilitate improved secretory functions in UECs.

One important function of the secretory endometrium is to prepare the uterus for embryo implantation. The endometrial receptivity array (ERA) is used clinically to help identify the WOI opening.^[^ ^12b^ ^]^ We compared the expression patterns of representative ERA genes following E_2_+P4+cAMP treatment and found that the ALI‐EnAos more closely recapitulated the defined WOI opening (Figure S7f, Supporting Information). Notably, in ALI‐EnAos, the WOI genes, *SPP1*, *GPX3*, *DEFB1*, and *ARG2—*whose upregulation is required for embryo implantation,^[^ ^12b^ ^]^ were significantly upregulated. Conversely, genes, including *BIRC1*, *SERPINA5*, *CRABP2*, and *ECM1*, which normally decline during mid‐secretory phase,^[^ ^12b^ ^]^ were significantly decreased in ALI‐EnAos (Figure 7f). These data indicate that ALI‐EnAos may provide a better model to study the mechanisms of human embryo implantation. The WNT signaling pathway is important for the formation of LE in vivo,^[^ ^25^ ^]^ we noted that noncanonical and canonical WNT signaling‐related genes in ALI‐EnAo epithelial cells were significantly upregulated (Figure 7g; Figure S7e, Supporting Information).

Together, our data show that ALI culture improves cell composition, anatomical structure, and gene expression patterns in EnAos by upregulating genes associated with polarity, ciliogenesis, decidualization, secretion, WNT signaling, and the WOI.

## 3 Discussion

The endometrium, the inner mucosal lining of the uterus, undergoes dynamic changes, including shedding, regeneration, and differentiation throughout each menstrual cycle.^[^ ^1^ ^]^ The endometrium is essential for a successful pregnancy as it is the site of implantation (luminal epithelium) and enables the subsequent nutrient exchange that supports the developing conceptus (glandular epithelium).^[^ ^13a^ ^]^ Although GLSs^[^ ^30^, ^47^ ^]^ or assembloids, formed by combining GLSs with EnSCs^[^ ^10a,b^ ^]^ have previously been established, these models are limited to the GLS alone and lack LELSs. In this study, we report a novel endometrial assembloid (ALI‐EnAo) with complete LELSs and GLSs; this ALI‐EnAo is formed by combining GLSs and EnSCs and using an improved MAC matrix and ALI culture method. By extensive comparative analyses, we have revealed that ALI‐EnAos closely recapitulate the features of the in vivo endometrium, including its anatomy, cell composition, hormone‐induced menstrual cycle changes, and gene expression profiles. Specifically, ALI‐EnAos recapitulate the gene expression patterns of the WOI and dynamic ciliogenesis, which are critical for embryo implantation. To our knowledge, this is the first organoid with a LE structure. The ALI‐EnAo model will be valuable for deciphering the mechanisms of embryo implantation, endometrial disease, and regeneration.

Using the assembloids derived from primary EnSCs and GLSs, we have revealed that EnSCs are essential for the generation of physiological GLSs, particularly for promoting GLS formation, regulating dynamic ciliogenesis induced by hormones, and improving gene expression patterns in EnECs. The gene expression patterns showed that different functions of EnSCs may be implemented by specific EnSC subpopulations in the endometrium. This in vitro model can therefore help to clarify the cellular interactions and functions of EnSC subpopulations in the endometrium during shedding, regeneration, and differentiation.

In mammals, the uterus arises from the intermediate mesoderm and gradually develops from the Müllerian ducts in the fetus. The endometrium then forms from the inner lining of the coeloms.^[^ ^33^ ^]^ The LE of the endometrium establishes the boundary to the uterine cavity and is remodeled during the menstrual cycle to become receptive to the implanting embryo. The particularity of the coelomic epithelium lies in the air‐liquid surface at birth. In our study, we found that the ALI‐cultured EnAos can generate an endometrium‐like structure with both a LE and GE. The LELS in the ALI‐EnAo was composed of columnar or cubic polar epithelium with strong proliferative abilities, confirmed by typical markers reported in previous results. However, the SC‐cultured surfacial layer cells did not have the same proliferation abilities as the ALI‐LE. Our model could therefore be used to better understand the mechanisms of LE generation, which may provide insight into the regeneration of the endometrium during menstruation or following injury.

Ciliated cells and their dynamic changes during the menstrual cycle are important for maintaining endometrial function. Although WNT‐activated transcription factors (such as *FOXJ1*)^[^ ^25^ ^]^ and the inhibition of NOTCH signaling^[^ ^18^ ^]^ are known to be important for ciliogenesis, the precise mechanisms of human ciliogenesis and the cilia dynamic changes remain unclear. In this study, we found that the ALI culture and EnSCs were crucial, both for ciliogenesis and dynamic cilia changes. Previous studies have shown that ALI culture facilitates epithelial differentiation.^[^ ^17a^ ^]^ Importantly, the ciliated cells and their pattern of dynamic changes accurately reflect those in the endometrium in vivo. Using our model, we were also able to identify potential genes involved in these processes (Figure 1f,g and Figure 7d). Following 18 passages, the EnSCs and GLSs still maintained similar formation abilities to the EnAOs. Combining this model with gene editing approaches will provide important insights into the functions of these genes during the regulation of dynamic ciliogenesis, similar to previous gene editing studies, which have successfully validated gene functions in intestinal organoids.^[^ ^48^ ^]^

In the human menstrual cycle, a pregnancy can only occur if embryo implantation happens within the WOI. Normally, the WOI occurs during the mid‐secretory phase of the menstrual cycle. Several morphological and molecular characteristics define the WOI, including the decidualization of EnSCs, reduced ciliation, optimal development of endometrial glands, and WOI gene expression, as represented by the endometrial receptivity array (Figure 7h). Our findings show that the ALI‐EnAos appropriately reproduce the implantation‐related signatures. Specifically, ALI‐EnAos on D4 of the Sec‐phase correspond to the WOI in vivo, which may enable ALI‐EnAos to be used as a model for studying implantation. However, there are some challenges associated with this, as the lack of fluid caused by the direct exposure of the EnAo to air is detrimental to embryo survival. To address this, improved strategies could potentially be used to coculture embryos. For example, the embryo could be embedded in a small amount of Matrigel and placed on the ALI model. We also found that the LE in the ALI model secreted a small amount of liquid, which may prevent the embryos from being exposed to air. Additionally, we noticed that the population of EnSCs was sparse in the ALI‐EnAos compared with in vivo EnSCs. This discrepancy suggests that the extracellular matrix in the study may be not optimal for supporting EnSC growth. For future studies, a more suitable extracellular matrix, such as hydrogel derived from decellularized tissue, will be required to better support EnSC growth and to improve EnAos structures and functions.

In conclusion, we have established a novel endometrial assembloid model with complete LE and GE structures. By incorporating EnSCs with organoids derived from patients, this innovative model may enable the modeling of different endometrial diseases that contribute to infertility and pregnancy failure and may facilitate the development of personalized and precise treatment plans for Assisted Reproductive Technology. Furthermore, this model will also be valuable for studying the complex human maternal‐fetal interaction.

## 4 Experimental Section

### Ethical Statement and Endometrial Sample Collection

This work was approved by the Medicine Ethics Committee of The First People's Hospital of Yunnan Province (KHLL2021‐KY069). Endometrial biopsies were collected in the First People's Hospital of Yunnan Province. The Medicine Ethics Committee of The First People's Hospital of Yunnan Province was composed of nine members, including lawyers, scientists, and clinicians with relevant expertise. The Committee evaluated the scientific merit and ethical justification of the study and conducted a full review of the donations and the use of these samples. All donors underwent an established protocol for obtaining informed consent, which occurred after the completion of a donor's clinical visit by an independent research coordinator. Healthy endometrial biopsy donors were from The First People's Hospital of Yunnan Province who sought for assisted reproductive technology due to oviduct factors or abnormal sperm of husband. All donor volunteers signed informed consents for voluntary donations of endometrium for human endometrium study. No financial inducements were offered for the donations.

The collected tissues were preserved in PBS and subsequently processed for cell isolation within 2–4 h. Donor demographics for the samples used in each experiment are detailed in Table S1 (Supporting Information). The donors were aged 20–35 years with regular menstrual cycling (3–7 d every 28–35 d) and negative serological tests for human immunodeficiency virus, hepatitis B virus, hepatitis C virus, and syphilis. Women with the following conditions were excluded from tissue donors: history of hormone use in the past two months, uterine pathology (endometriosis, leiomyoma, or adenomyosis; bacterial, fungal, or viral infection), or polycystic ovary syndrome.

Endometrial biopsies were obtained from endometrial tissues during the proliferative phase (11th to 14th day in the menstrual cycle) and secretory phase (6 to 10 days after the pre‐ovulatory luteinizing hormone surge).

### Primary Endometrial Cell Culture

Cell cultures were all incubated in a humidified incubator at 37 °C. Centrifugation and incubation steps were performed at room temperature unless otherwise indicated. The isolation and expansion of endometrial gland‐like structures (GLS) from endometrial biopsies were performed as previously described protocol.^[^ ^6a^ ^]^ Briefly, the obtained endometrial biopsies were finely minced with a scalpel into ≈0.5 mm^3^ cubes, collected in a 50 mL centrifuge tube with 10 mL of isolation medium (RPMI 1640 medium (Thermo Fisher Scientific, 11 875 093) containing 1.25 U mL^−1^ of Dispase II (Sigma–Aldrich, D4693), 0.4 mg mL^−1^ of collagenase V (Sigma–Aldrich, C‐9263) and 10% fetal bovine serum (FBS, BioInd, 04‐001‐1A), and gently shaken at 37 °C for 30–60 min until a large number of glands were observed under a microscope. After that, the supernatant was filtered through one or more 70 µm cell strainers (Corning, CLS431751), and washed several times with RPMI 1640 medium. To obtain endometrial stromal cells (EnSCs), the filtered suspension was collected and the supernatant was removed after centrifugation. The cell masses were resuspended with DMEM (BasalMedia, L110KJ) + 10% FBS (BioInd, 04‐001‐1A) + LAA (Sigma–Aldrich, A4544) culture medium and seeded into dishes for 2D culture. EnSC decidualization was induced by 10 nm β‐oestradiol (E_2,_ Sigma–Aldrich, E8875), 1 µm progesterone (P4, Sigma–Aldrich P0130) and 0.5 mm 8‐bromoadenosine 3′, 5′‐cyclic monophosphate (cAMP, Sigma B7880) for 96 h, and confirmed by the expression of IGFBP1 and PRL by Real‐time qPCR.

The glands that had been captured in the filter were backwashed from the filter membrane, centrifuged, and then resuspended in pre‐chilled 70% Matrigel (Corning, 356 231) diluted with Advanced DMEM/F12 (Gibco, 12 634 010). Twenty five microliters of the mixture per well was transferred into a 48‐well plate (Costar, 3548), placed at 37 °C for 30 min, and then covered with 250 µL of Expansion Medium (ExM).^[^ ^6a^ ^]^ After two passages, to mimic proliferative and secretory glands, GLSs were treated with 10 nm E_2_ or subsequent 1 µm P4 and 0.5 mm cAMP treatment (Figure S1d, Supporting Information).

When GLSs were frozen, they were mixed with a Recovery cell culture freezing medium (Thermo Fisher Scientific, 12648‐010) into freezing tubes after the last centrifugation, and stored in liquid nitrogen.

Passage 2–5 EnSCs and Passage 3–9 GLSs were used for subsequent experiments. Cells from different passages yielded similar results.

### Assembly of EnAos and Improvement of Culture Matrix

To assemble endometrial assembloid (EnAo), EnSCs and EnECs with different ratios (1:0, 1:1, 1:2, 1:3, and 1:4) were resuspended with ice‐cold extracellular matrixes (ECMs) at a ratio of 1:20 (cell pellet: matrix). To screen the culture matrix, three different matrixes were performed: Matrigel (Corning, 356 231), COLI (Sigma–Aldrich, 5074‐35mL), and MAC (Matrigel: COLI: Advanced DMEM/F12 = 1:1:1 (v/v)). Cell pellets were kept on ice until plating. The suspension was aliquoted in 25 µL volumes using ice‐cold pipette tips into a 48‐well plate and placed in the cell culture incubator for 45 min. The ExM medium was overlaid and supplemented with 10 nm E_2_ after 24 h. The medium was refreshed every 48 h. After 4 days of E_2_ treatment, EnAos were collected for subsequent assessment of the culture matrix.

### Measurement of Tissue and ECMs Stiffness

EnAos‐embedded ECMs and endometrium biopsies were collected and measured by a compression test using a Nanoindenter (Piuma, Optics11). Endometrium biopsies and EnAos from three donors were repeated for statistical analysis. At least ten points were detected per sample.

### Construction of ALI‐EnAos and SC‐EnAos Model and Hormonal Stimulation

EnAos were suspended in the MAC at a ratio of 1:20 (cell pellet: matrix) and maintained chilled until plating. The suspension was injected into the upper chamber of the Transwell (Sigma, CLS3470‐48EA) in 45 µL volumes using ice‐cold pipette tips, and it was left to cure for an hour in the cell culture incubator. Solidified MAC gel forms a layer ≈1 mm thick. Then GLSs from same donor source in a 48‐well plate were removed from Matrigel by pipetting several hundred times and broke apart into fragments. Then these GLS fragments were resuspended with ExM (100 µL) and added into the upper chamber of the Transwell. GLS on top of MAC per upper chamber of Transwell contained ≈2–4 × 10^4^ EnECs. ExM medium (400 µL) was added to the below chamber.

The liquid was removed from the upper chamber and the below chamber was maintained with 400 µL of medium for air–liquid interface (ALI) culture. The medium in the below chamber was changed every 48 h. For submerged culture (SC), the medium in the upper chamber was retained and changed every 48 h. The time to start the ALI culture depends on experiment demands (Figure 3b and Figure 4a).

Hormonal stimulation of EnAos in the ALI or SC culture was performed with 10 nm E_2_, 1 µm P4, and 0.5 mm cAMP. To mimic the proliferative endometrium, we treated EnAos with E_2_ for 4 days and collected them for further examinations on D4 and D15 after ALI or SC culture. To mimic secretory endometrium, EnAos were treated with E_2_ for 4 days, followed by P4 and cAMP for 4 days, and collected them for further examinations on D4 and D15 after ALI or SC culture.

GLSs and EnSCs from three different donors were used in the experiment.

### Frozen Section Staining and Taking Photographs

The samples were fixed in 4% paraformaldehyde for 4 h. For tissues, dehydration was performed with a 10%, 20%, and 30% concentration gradient of sucrose followed by embedding using OCT (SAKURA, 4568). EnAo or GLSs were dehydrated with 20% sucrose solution for 40 min and then separated from Transwell membrane carefully. Then the samples were embedded with OCT. After frozen, frozen sections of 5 µm were cut with a freezing microtome and collected on adhesive slides. Before staining, the slides were washed by PBS three times for 10 min each to clear OCT, and permeabilized and blocked with 100–200 µL 3% BSA containing 0.4% Triton X‐100 for 3–4 h at room temperature or overnight at 4 °C. The section was washed three times with 0.05% Tween‐20. Primary antibodies were incubated overnight at 4 °C (Antibody details are presented in Table S2, Supporting Information). Secondary antibodies were labeled using 488/568/647 (1:500) and DAPI (1:1000) at room temperature for 2 h. Sections were washed three times in PBS for 10 min each. A drop of 10–20 µL 50% glycerol was added and the slides were blocked with coverslips. The pictures were taken with Leica SP8 or Leica X confocal microscope. The super‐resolution images were taken by Zeiss Elyra 7 with Lattice SIM^2^.

### RNA Extraction and Gene Expression Analysis

After 4 days of decidual treatment, EnSCs were collected for gene expression analysis. Then EnSCs were treated with TrypLE for 5 min at 37 °C and dissociated into single cells by pipetting up and down gently. Total RNA was extracted using TRIzol Reagent (Thermo Fisher Scientific, 15 596 018) according to the manufacturer's protocol. RNA concentration and purity were determined using Nanodrop Spectrophotometers (Thermo Scientific, ND2000). RNA (2 µg) from each sample was subjected to cDNA synthesis using the PrimeScript RT reagent Kit (Takara, RR047A). Real‐time qPCR reactions were set up in triplicate with ChamQ Universal SYBR qPCR Master Mix (Vazyme, Q711‐02) and run on CFX Duet Real‐Time PCR System (Bio‐Rad). Quantification of gene expression was based on the Ct (Cycle threshold) values with β‐Actin served as the internal control. The sequences of primers are: PRL forward: 5′‐TGT CCC ACT ACA TCC ATA A‐3′, PRL reverse: 5′‐TAC AGA GGC TCA TTC CAG‐3′, IGFBP1 forward: 3′‐AGT ACC TAT GAT GGC TCG‐3′, IGFBP1 reverse: 5′‐ACA CTG TCT GCT GTG ATA AA‐3′, β‐Actin forward: 5′‐CAT GTA CGT TGC TAT CCA GGC‐3′, β‐Actin reverse: 5′‐CTC CTT AAT GTC ACG CAC GAT‐3′. Quantification was performed using the 2^−ΔΔCt^ method after normalization against controls.

For RNA sequencing, the 2 × 150 bp paired‐end libraries were sequenced with Illumina NovaSeq 6000. Library construction and sequencing were performed by Annoroad Gene Technology (http://www.annoroad.com/). Reads mapping and transcript expression level quantification were carried out utilizing the workflows of HISAT2, StringTie, and the R package DESeq2 (v1.30.1). In a nutshell, the HISAT2 software (v2.2.1) program was used to align paired‐end clean reads against the human reference genome (hg38). Transcript assembly, GTF document mergence, and transcript abundance estimation were performed using StringTie (v2.1.1). The read count information for each transcript was taken from the coverage values calculated by StringTie using the prepDE.py Python script. Following the prepDE.py results, DESeq2 was used to analyze the differential expression of genes (DEG) (v1.30.1). Genes with *p* Value <0.05 and log2 (FoldChange) >1 were regarded as DEGs. R package “pheatmap” (v1.0.12) was used for heat mapping of selected genes.

### Dissociation of EnAos for Single‐Cell Analysis

After removed from the Transwell chambers, the EnAos were incubated in 0.5 mg mL^−1^ collagenase I (Yeason Biotechnology, 40507ES60) at 37 °C water bath for 45 min and shaken gently at regular intervals to degrade the gel mixture. Samples were centrifuged at 1000 rpm for 5 min and washed with Advanced DMEM/F12 medium. After centrifugation again, the cells were incubated in a 37 °C water bath with TrypLE suspending for 60 min. The cell clumps were broken up by manual pipetting gently and left to dissociate into single cells. Cells were then suspended in PBS containing 0.1% bovine serum albumin (BSA). This suspension was passed through a 70 µm cell strainer. Another 1 mL of PBS containing 0.1% BSA was passed through the cell strainer to clean the filter membrane. Cell viability was assessed on a TC20 automated cell counter (Bio‐Rad) using Trypan Blue (#1 450 013, Bio‐Rad). Cell viability was higher than 95%.

### Single Cell RNA Sequencing and Data Analysis

Single cells suspended in PBS containing 0.1% BSA were loaded in 10x Genomics Chromium within 30 min after dissociation. 10x Genomics v3.1 libraries were prepared according to the manufacturer's instructions. Libraries sequencing were performed by Annoroad Gene Technology (http://www.annoroad.com/) on an Illumina NovaSeq 6000 with 150‐bp paired‐end sequencing.

The sequencing data was mapped to GRCh38 human reference genome through CellRanger (v5.0.1) and the default number of cells was 10 000 to generate a digital gene expression matrix. The data from all samples were read into the R package Seurat (v4.2.0) for quality control and downstream analysis of the single‐cell RNA‐seq data. All functions were run with default parameters. Cells were filtered based on unique molecular identifiers (UMIs), the number of expressed genes, and the expression mitochondria gene fraction (detailed in Table S2, Supporting Information). First, data from each sample was normalized separately using the NormalizeData function and scaled with the ScaleData function implemented in the Seurat package. Then, data were integrated across each sample using the Seurat functions FindIntegrationAnchors and IntegrateData, based on 30 dimensions and 2000 anchor features. The integrated data was scaled by unit variance and zero mean. The dimension of data was reduced by principal component analysis (PCA). Finally, cell types were defined based on gene expression and clusters identified through the FindClusters function. Cluster‐specific marker genes were identified by running the FindAllMarkers function in Seurat. The FindMarkers function was used to identify DEGs between two cell clusters. Genes with *p* Value <0.05 and log2 (FoldChange) >1 were regarded as DEGs. The R package clusterProfiler (v3.18.1) was utilized to perform GO enrichment analysis with the DEGs in each cluster or subset. Enrichments of GO terms were visualized using the ggplot2 (v3.3.6) package in R (All the information related to DEGs and GO terms is in the Supporting Information). Monocle (v2.18.0) was used to perform a trajectory analysis for the cell type under consideration. The cells were then arranged in pseudotime using Monocle after applying a dimensionality reduction to the data. Data from Wang et al.^[^ ^19^ ^]^ (10x Chromium scRNA‐seq data of both the proliferative and secretory phase endometrium) were integrated with our data to compare the transcriptomics of EnAos with human endometrium. The LE/GE populations in vivo shown in Figure 6a were based on the definitions made by authors in the reference according to their in vivo transcriptome and staining.^[^ ^19^ ^]^ Based on the integrated data, the ALI‐EnAo datasets were then subsetted into 25 clusters, which could be annotated into LE‐ or GE‐like population based on the expression of their markers. The UMAP distribution of LE/GE‐like populations in ALI‐EnAo was presented. The main signaling inputs and outputs in stromal and epithelial cells were evaluated using CellChat (v1.1.3). The function netVisual_circle was used to display the communication strength of designated receptor‐ligand pairs between cells.

### Statistical Analysis and Reproducibility

Single‐cell RNA data was normalized separately using the NormalizeData function before statistical analysis. Data were checked for normal distribution and equal variances before each parametric statistical test was performed. All quantification data were shown as the means ± SEM. At least three independent experiments were performed (Figure legends indicate the number of independent experiments and statistical subjects performed in each analysis). Image J software was used to measure the diameters of gland‐like structure (GLS). The diameter of each GLS was determined by the average of the longest inner diameter and the shortest inner diameter. When counting the number of GLS, GLS with a diameter >10 µm was counted. Wilcoxon test was used to calculate the statistical significance of gene expression in scRNA‐seq. Chi‐square test was used to analyze the percentage of cells or GLS subtypes with the SPSS26 software. GraphPad Prism 8 software and unpaired Student's *t*‐test (two‐sided testing) were used to perform the remaining statistical analyses of bright field, Real‐time qPCR, and immunofluorescence staining. When counting Ac.α‐tubulin^+^ cells, only the cells with cilia‐like bulge staining positive at the apical cell membrane were taken into quantification. Only values of *p* < 0.05 were considered statistically significant. ^*^ *p* < 0.05, ^**^ *p* < 0.01, ^***^ *p* < 0.001, ^****^ *p* < 0.0001. All experiments reported in this study were reproduced with similar results using independent samples (tissues and cells) from at least three donors.

## Conflict of Interest

The authors declare no conflict of interest.

## Author Contributions

J.T., J.Y., and T.C. contributed equally to this work. T.L. and Y.M. initiated the project. T.L. designed the experiments, organized, and supervised the entire project. T.L., J.T., and J.Y. wrote the manuscript. J.T. and J.Y. performed experiments and analyzed data. T.C. and Y.Y. performed bulk RNA and single‐cell sequencing analysis. N.L. performed 10× single‐cell library construction. Y.L. and X.L. collected the clinical samples. E.D. assisted in part of molecular experiments. H.T. assisted in part of the data analysis.

## Supporting information

Supporting Information

Supplemental Table 1

Supplemental Table 2

Supplemental Table 3

Supplemental Table 4

Supplemental Table 5

Supplemental Table 6

Supplemental Table 7

Supplemental Table 8

Supplemental Table 9

Supplemental Table 10

## Data Availability

The data that support the findings of this study are available from the corresponding author upon reasonable request.
