# Supplementary material for: Generation of Human Endometrial Assembloids with a Luminal Epithelium using Air–Liquid Interface Culture Methods
Source: Adv Sci (Weinh). 2023 Aug 27;10(30):2301868. doi: 10.1002/advs.202301868 (PMC10602567; doi:10.1002/advs.202301868)
Supplement: Supplementary file 1 — Supporting Information [file ADVS-10-2301868-s009.pdf]

ADVANCED SCIENCE
Open Access

Supporting Information

for *Adv. Sci.*, DOI 10.1002/advs.202301868

Generation of Human Endometrial Assembloids with a Luminal Epithelium using Air–Liquid Interface Culture Methods

Jiwen Tian, Jie Yang, Tingwei Chen, Yu Yin, Nan Li, Yunxiu Li, Xingyu Luo, E Dong, Haoyang Tan, Yanping Ma and Tianqing Li**

Supporting Information

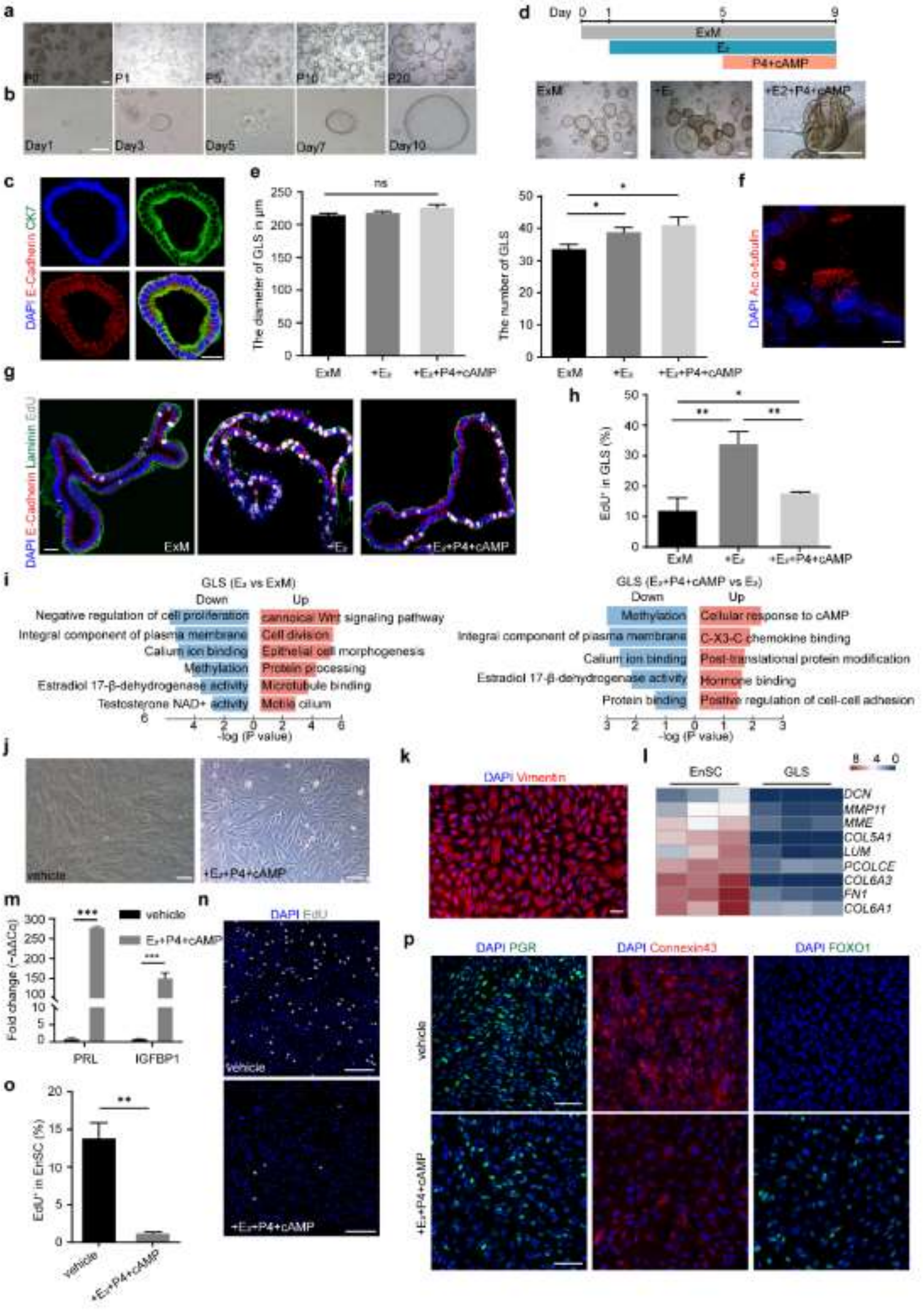

Supplementary Figure 1

Isolation and characterization of endometrial glands and primary endometrial

stromal cells (EnSCs), relative to Figure 1.

a. Representative phase-contrast images of primary isolated endometrial glands (P0) on D0 and expanded glands-like structures (GLSs) over passaging on D3 in Expansion Medium (ExM). Endometrial glands from 15 donors were isolated and similar results were obtained. Scale bars: 100 μm.

b. A representative GLS generated from a single epithelial cell. Gland epithelium from 3 representative donors were assessed, yielding similar results. Scale bar: 50 μm.

c. Representative staining of E-cadherin and cytokeratin-7 (CK7) in GLSs. Gland epithelium from 3 representative donors were assessed, yielding similar results. Scale bar: 20 μm.

d. Protocols (top) and representative phase-contrast images (bottom) of GLSs that were cultured in the control medium (ExM), E_2, or E_2+P4+cAMP. Scale bars: 100 μm.

e. The diameters in μm and numbers (per 10X field of view) of GLS in the ExM or treated by hormones on D9 (Supplementary Figure 1d). The results were obtained from 3 donors.

f. Representative cilium (acetylated α-tubulin, Ac.α-tubulin) in epithelial cells by E_2 treatment on D9 (Supplementary Figure 1d). Each donor cells repeated twice (*N*=3 donors). Scale bar: 10 μm.

g. Representative staining of E-cadherin, Laminin and EdU in GLS without or with the treatment of hormones. Scale bars: 20 μm.

h. Quantification of EdU^+ cells in GLSs without or with the treatment of hormones on

D9 (Supplementary Figure 1d). The results were obtained from 3 donors.

i. Representative gene ontology (GO) of the differentially expressed genes (DEGs) in E_2-treated vs. control (ExM) GLSs (left), and E_2+P4+cAMP-treated vs. E_2-treated GLSs (right). Bulk RNA-seq data of samples were collected on D9 of the protocol in Supplementary Figure 1d. Blue, down-regulated GO terms; Red, up-regulated GO terms. The results were obtained from 3 donors.

j. Representative contrast-phase images of EnSCs (Passage 2-5) showing that EnSCs exhibited typical fibroblast morphology (vehicle, no hormones treatment) and occurred morphological changes after decidualization (E_2+P4+cAMP). Scale bars: 100 μm.

k. Representative staining of mesenchymal marker Vimentin in EnSCs. Scale bar, 20 μm. Experiments were repeated with EnSCs from endometrium samples of 3 donors.

l. Heatmap of EnSC marker genes in EnSCs and GLSs after 3 days culture without hormone treatment from 3 different donors. Scale represents as the log2 (data+1).

m. Decidualization treatment (E_2+P4+cAMP) significantly upregulated marker genes prolactin (PRL) and insulin like growth factor binding protein 1 (IGFBP1) in EnSCs by Real-time qPCR analysis. Each donor cells repeated twice (N=3 donors). Vehicle, no hormones treatment.

n. Decidualization of EnSCs is associated with decreased EdU^+ cells. Scale bars: 200 μm.

o. Quantification of EdU^+ EnSCs in the no hormones treatment culture (vehicle) and decidualization culture (E_2+P4+cAMP). EdU^+ cells exhibited a significant decrease

after decidualization. (*N*= 3 donors).

p. Representative staining of nuclear PGR, intercellular junction marker Connexin43 and nuclear FOXO1 in EnSCs under no hormones treatment culture (vehicle) and decidualization culture (E_2+P4+cAMP). Experiments were repeated with EnSCs from endometrium samples of 3 donors. Scale bars: 100 μm.

e, h, m, o, data are presented as mean ± SEMs; Two-sided unpaired Student's t test was used to perform the statistical analyses of staining, bright field and Real-time qPCR; $*P \leq 0.05$; $**P \leq 0.01$; $***P \leq 0.001$; $****P \leq 0.0001$; ns, no significance.

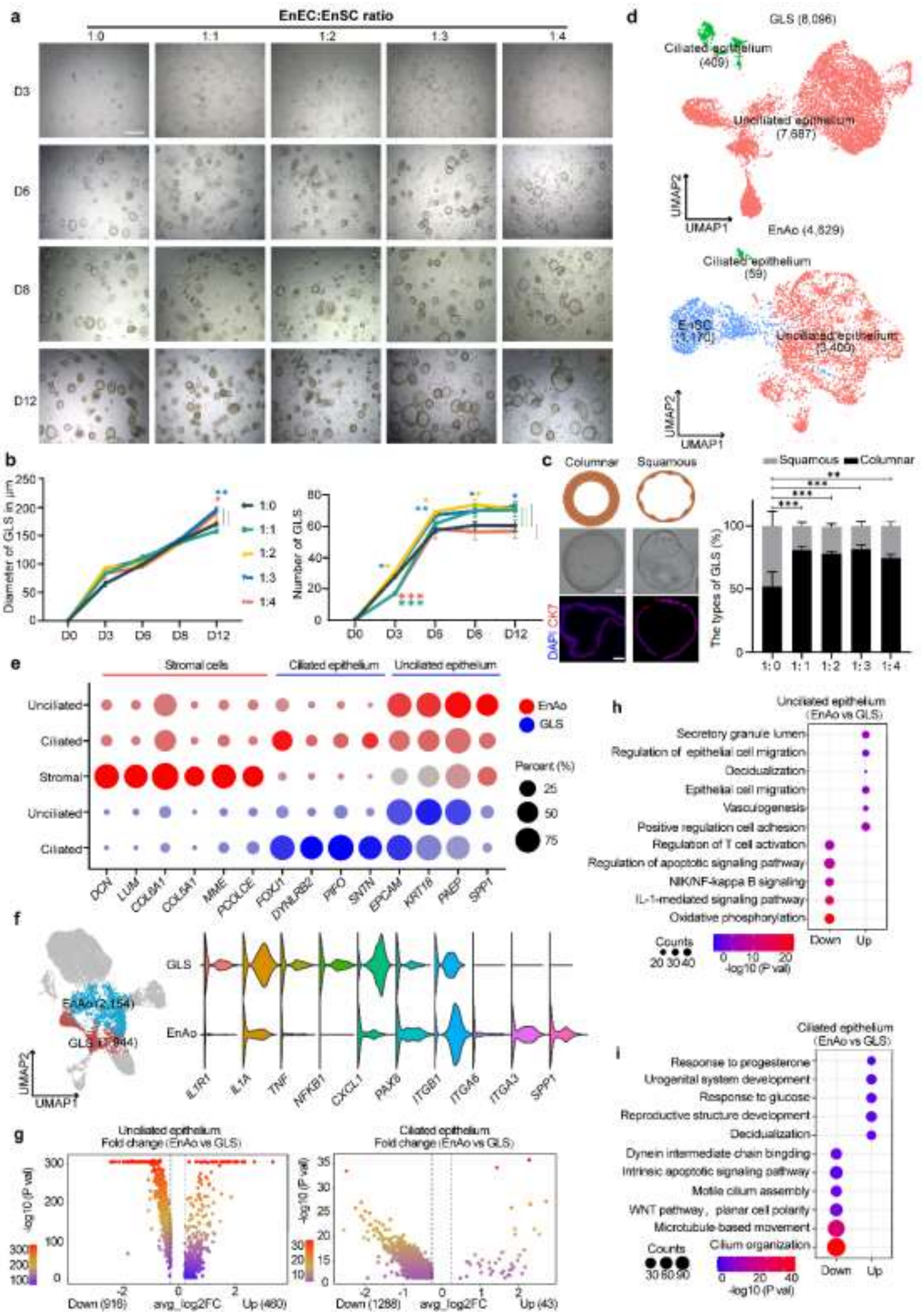

Supplementary Figure 2.

Endometrial stromal cells (EnSCs) contribute to glandular phenotypes in Matrigel, relative to Figure 1

a. Representative phase-contrast images of different endometrial epithelial cells (EnEC): EnSC ratio coculture over culture. Scale bars: 250 μm.

b. Diameter (left) and number (per 10X field of view) (right) of gland-like structure (GLS) in different EnEC:EnSC ratio coculture over culture. Five views were quantified each experiment. The comparisons at each time point (D3, D6, D8, D12) were performed between stromal-free group (1: 0) and different ratio of EnEC:EnSC groups (1:1, 1:2, 1:3, 1:4), respectively. The comparison results of different time point were presented at the top of each time points on the X-axis, and different colors of "*" were used to represent corresponding EnEC:EnSC ratio groups.

c. Characterization (left) and quantification (right) of columnar- and squamous-type GLS in different EnEC:EnSC ratio coculture on Day 12. The GLS subtypes were identified according to epithelial cell morphologies and nuclear arrangement. At least more than 100 GLSs were quantified each experiment. Scale bars: contrast phase, 50 μm; immunofluorescence, 25um.

d. The distribution of different cell types in GLSs and EnAos at sec-phase after E_2+P4+cAMP treatment (as protocol of Supplementary Figure 1d) by UMAP analysis.

e. Dot plots of some representative genes specific for each subtype in GLSs and EnAos.

f. The distribution and gene expression patterns in cell subpopulations in GLSs or EnAos.

g. Volcano plots of DEGs in unciliated cells (left) and ciliated cells (right) between

EnAos and GLSs.

h. Representative GO terms of downregulated or upregulated in unciliated cells of EnAos, compared to GLSs.

i. Representative GO terms of downregulated or upregulated in ciliated cells of EnAos, compared to GLSs.

b, c. Data are presented as means ± SEMs; Chi-square test was used to analyze percentage of GLS subtypes and two-sided unpaired Student's t test was used to perform the statistical analyses of bright field; *$P \leq 0.05$; **$P \leq 0.01$; ***$P \leq 0.001$; ****$P \leq 0.0001$; ns, no significance. Repeated experiments from 3 donors were assessed, yielding similar results.

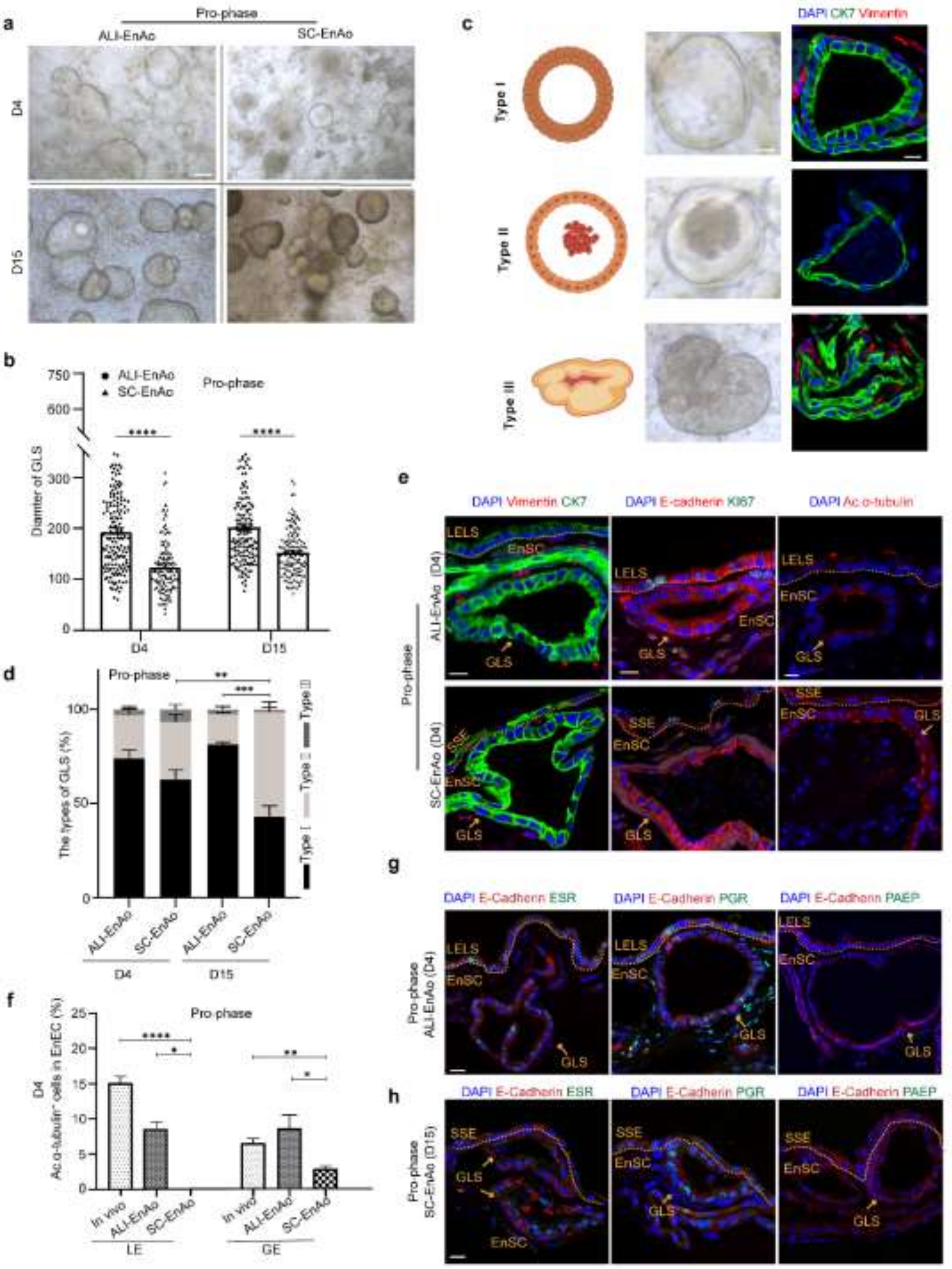

Supplementary Figure 3. Air-liquid interface culture (ALI-cultured) EnAos mimic proliferative endometrium during menstrual cycle, relative to Figure 3.

a. Representative images of ALI-EnAos and submerged culture (SC)-EnAos on D4 and D15. Scale bar: 100 μm.

b. The diameter of GLS in ALI-EnAos and SC-EnAos on D4 and D15. At least more

than 180 GLSs were quantified each experiment. Repeated experiments from 3 donors were assessed, yielding similar results.

c. Definition and characterization of three GLS subtypes. Scale bars: contrast-phase, 100 μm; staining, 25 μm

d. Quantification of three subtypes of GLS in ALI-EnAos and SC-EnAos on D4 and D15.

e. Representative staining of endometrial markers in ALI-EnAos and SC-EnAos on D4. CK7 for EnECs; Vimentin for EnSCs; KI67 for cell proliferation; E-cadherin for epithelium; and Ac.α-tubulin for ciliated cells. Scale bars: 25 μm.

f. Quantification of Ac.α-tubulin^{+} cells on LE and GE in endometrium, ALI-EnAos and SC-EnAos on D4.

g. Representative staining of E-cadherin, estrogen receptor (ESR), progesterone receptor (PGR) and progestogen associated endometrial protein (PAEP) in ALI-EnAo on D4. Scale bars: 25 μm.

h. Representative staining of E-cadherin, ESR, PGR and PAEP in in SC-EnAo on D15. Scale bars: 25 μm.

d, f, all data were obtained based on 3 different donors. **b**, **d**, **f**, data are presented as means ± SEMs; Chi-square test was used to analyze percentage of GLS subtypes and two-sided unpaired Student's t test was used to perform the statistical analyses of staining and bright field; *P ≤ 0.05; **P ≤ 0.01; ***P ≤ 0.001; ****P ≤ 0.0001; ns, no significance. LELS, luminal epithelium-like structure; GLS, gland-like structure; SSE, simple squamous epithelium.

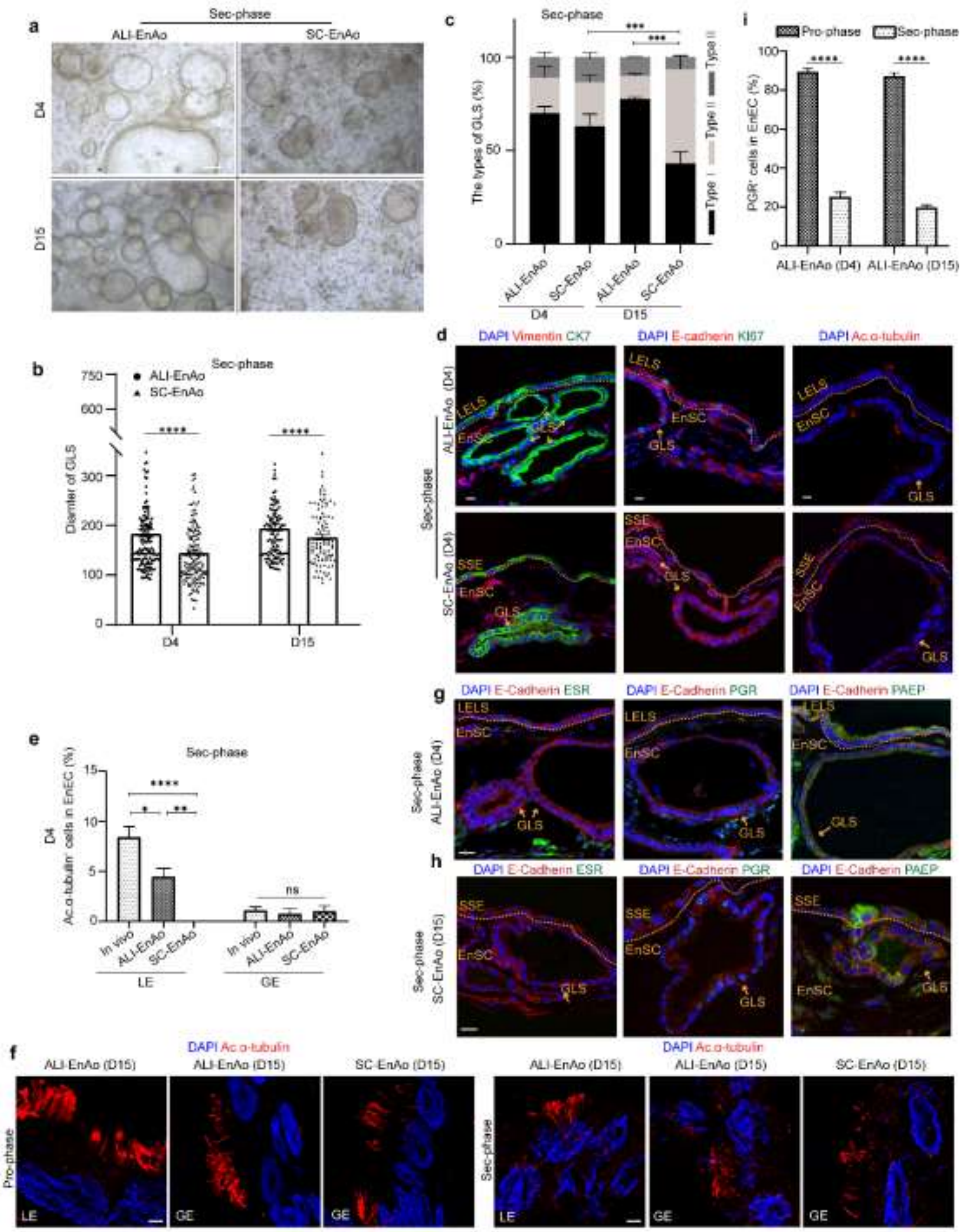

Supplementary Figure 4. ALI-cultured EnAos mimic secretory endometrium during menstrual cycle, relative to Figure 4.

a. Representative contrast-phase images of ALI-EnAos and SC-EnAos on D4 and D15. Scale bars: 100 μm.

b. The diameter of GLS in ALI-EnAos and SC-EnAos on D4 and D15. At least more

than 180 GLSs were quantified each experiment. Repeated experiments from 3 donors were assessed, yielding similar results.

c. Quantification of three subtypes of GLS in ALI-EnAos and SC-EnAos on D4 and D15.

d. Representative staining of endometrial markers in ALI-EnAos and SC-EnAos on D4. CK7 for EnECs; Vimentin for EnSCs; KI67 for cell proliferation; E-cadherin for epithelium; and Ac.α-tubulin for ciliated cells. Scale bars: 25 μm.

e. Quantification of Ac.α-tubulin^{+} cells on LE and GE in endometrium, ALI-EnAos and SC-EnAos on D4.

f. Representative staining cilia morphology under a super resolution system to show structured illumination images in ALI and SC on D15 across different phases. Scale bars: 2 μm.

g. Representative staining of E-cadherin, ESR, PGR and PAEP in ALI-EnAos on D4. Scale bars: 25 μm.

h. Representative staining of E-cadherin, ESR, PGR and PAEP in SC-EnAos on D15. Scale bars: 25 μm.

i. Quantification of PGR^+ cells on EnECs in ALI-EnAos during phases.

c, **e**, **i**, all data were obtained based on 3 different donors. **b**, **c**, **e**, **i**, data are presented as means ± SEMs; Chi-square test was used to analyze percentage of GLS subtypes and two-sided unpaired Student's t test was used to perform the statistical analyses of staining and bright field; $*P \leq 0.05$; $**P \leq 0.01$; $***P \leq 0.001$; $****P \leq 0.0001$; ns, no significance. LELS, luminal epithelium-like structure; GLS, gland-like structure; SSE, simple squamous epithelium.

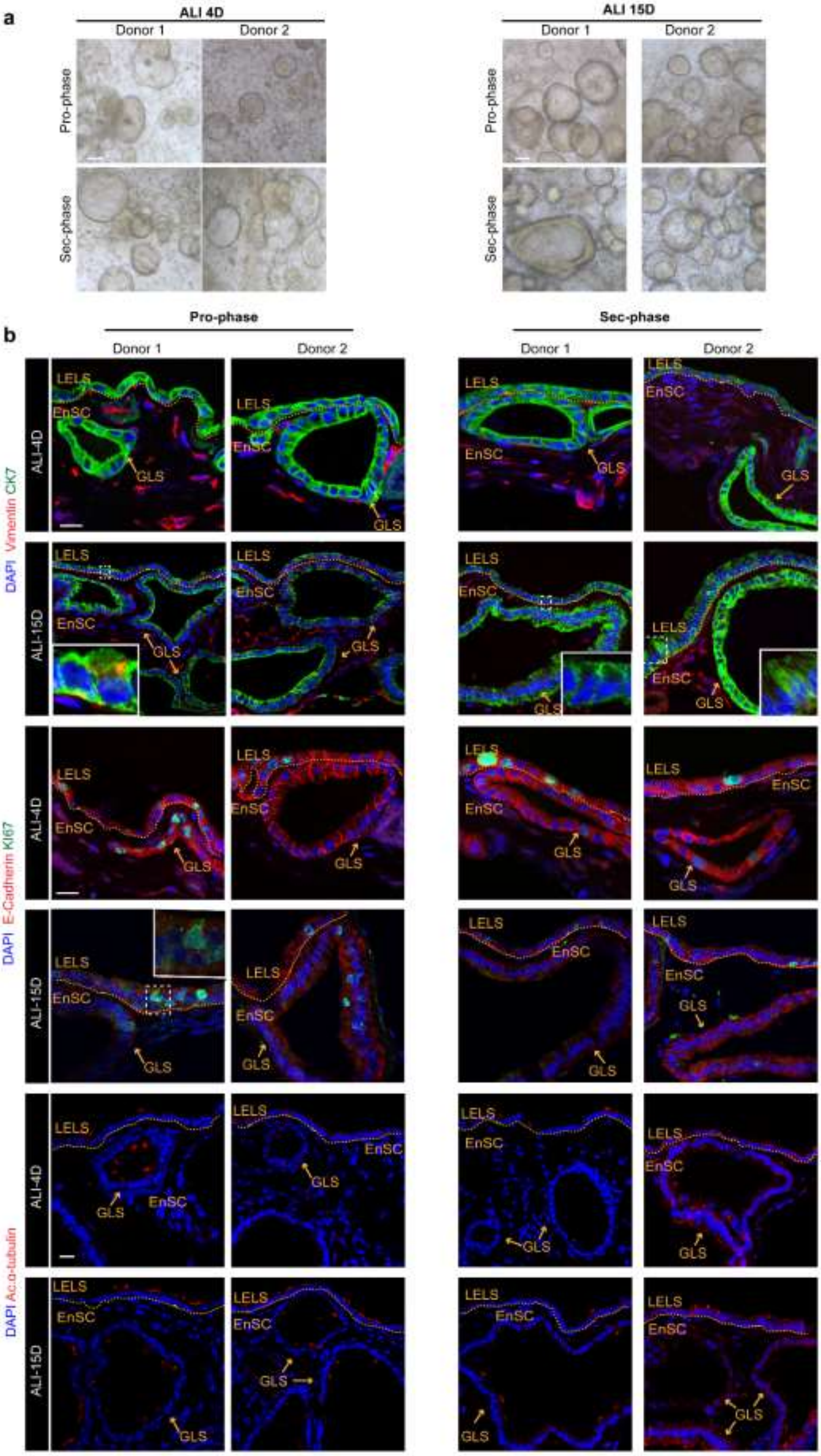
a
ALI 4D
ALI 15D
Donor 1
Donor 2
Pro-phase
Sec-phase
b
Pro-phase
Sec-phase
ALI-4D
ALI-15D
DAPI Vimentin CK7
DAPI E-Cadherin KI67
DAPI Ac-α-tubulin
LELS
EnSC
GLS

Supplementary Figure 5

The ALI-EnAos were reproducibily generated from other donor samples, relative to Figure 3 and 4.

The ALI-EnAos were successfully generated using additional two different donor samples. Quantification of staining were showed in Figure 3-4 and Supplementary Figure 3-4.

a. Representative images of ALI-EnAos on D4 and D15 during cycle. Scale bars: 100 μm.

b. Representative staining of endometrial cell markers in ALI-EnAos on D4 and D15. Scale bars: 25 μm. LELS, luminal epithelium-like structure; GLS, gland-like structure.

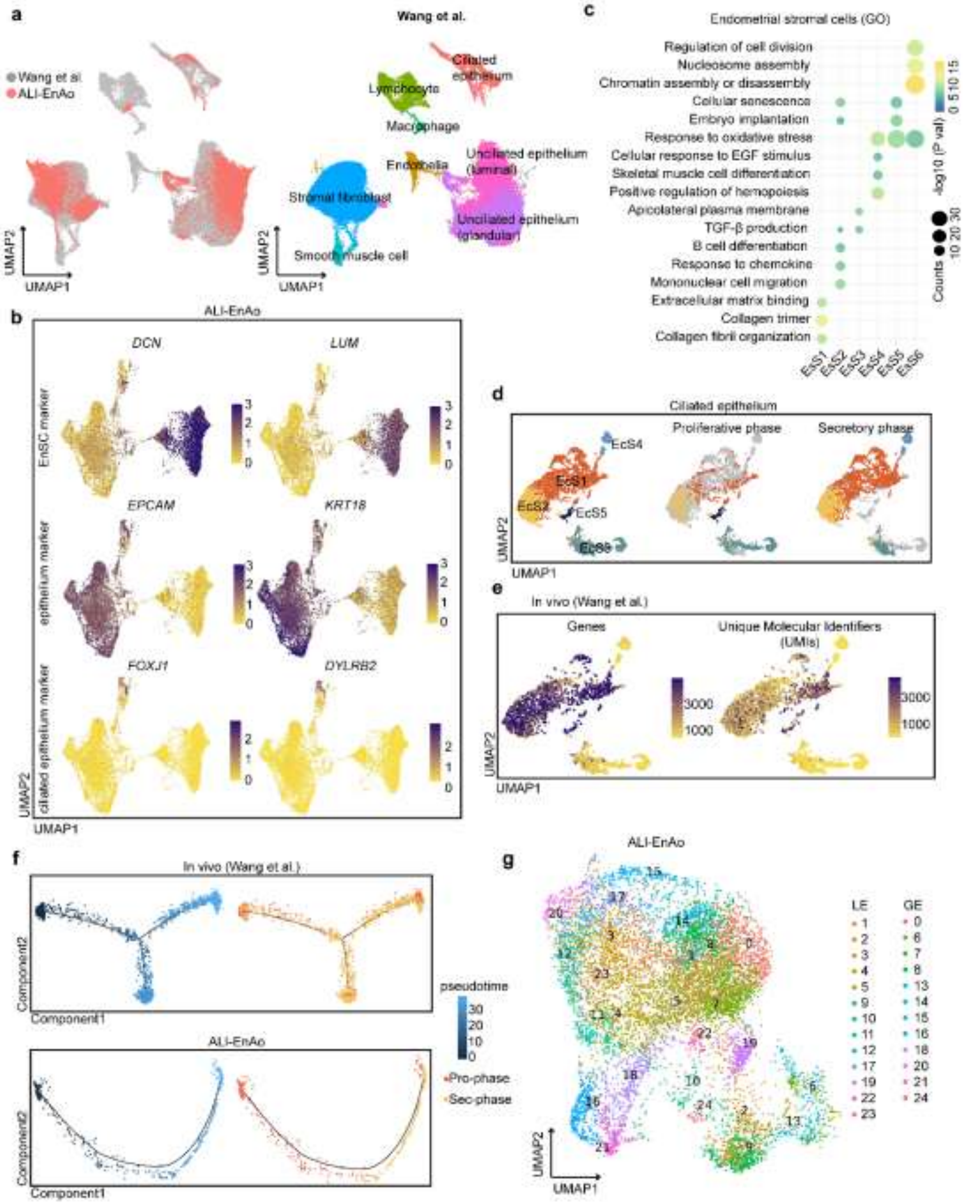

Supplementary Figure 6

ALI-EnAos possessing similar transcriptome as endometrium, relative to Figure 5.

a. UMAP analysis of integrated scRNA-Seq from ALI-EnAo (both Pro- and Sec-phase) and human endometrium across menstrual cycle (all the cell types from the

whole Pro- and Sec-phase).[19]

b. Cells in ALI-EnAos were annotated into three cell types according to the cannonical EnSC markers (*DCN* and *LUM)*, EnEC markers (*EPCAM* and *KRT18)* and ciliated cells markers (*FOXJ1* and *DYNLRB2*).

c. Representative GO terms corresponding to EnSC subpopulations.

d. Distribution of ciliated cell subpopulations during menstrual cycle.

e. Genes and unique molecular identifiers (UMIs) in ciliated population in endometrium in vivo.

f. Pseudotime showing the differentiation trend of ciliated cell across different phases between in vivo and ALI-EnAos.

g. The ALI-EnAo datasets were subsetted into 25 clusters, which were further annotated into LE- or GE-like subpopulation based on the expression of their markers, relative to Figure 6a-b.

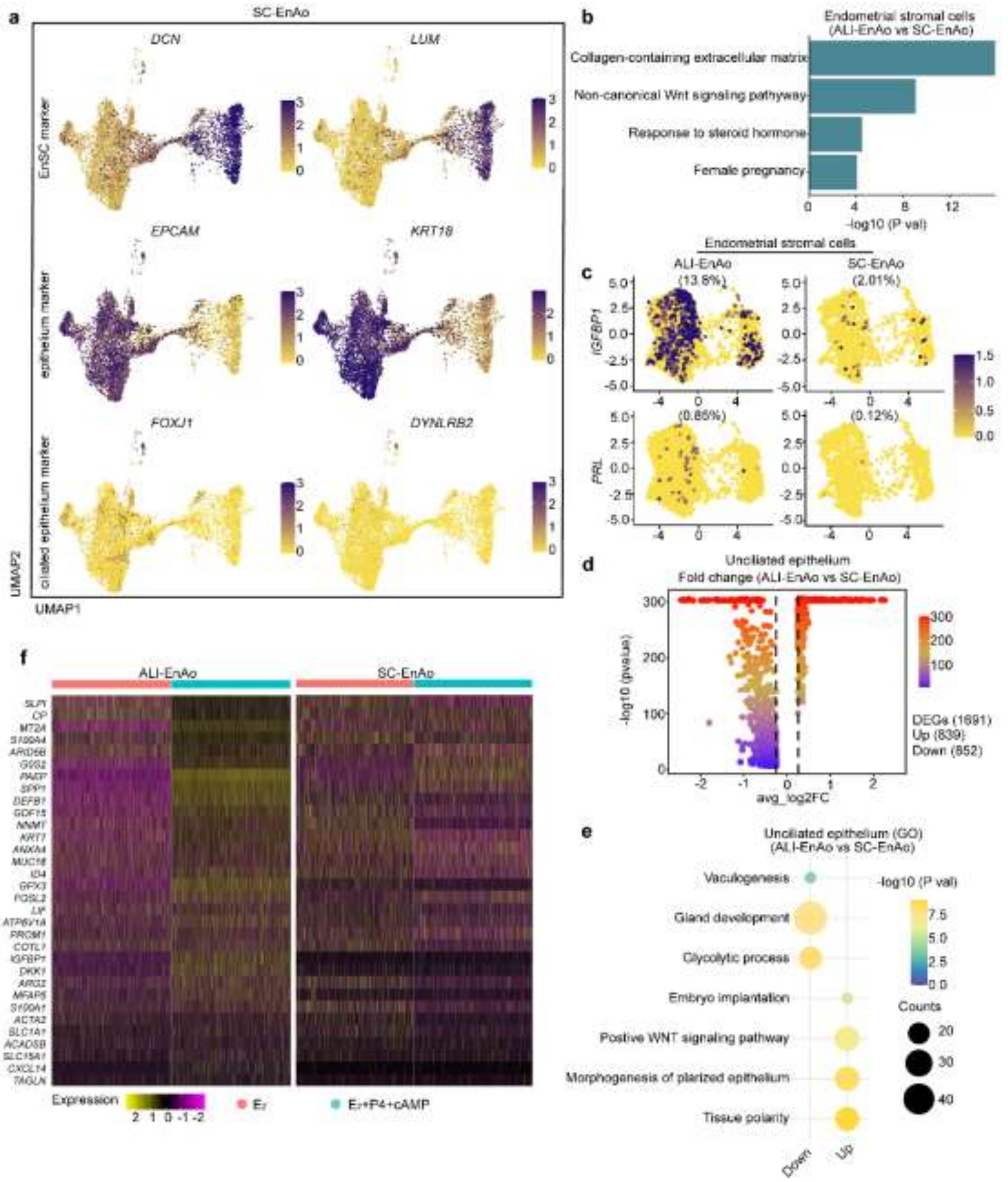

Supplementary Figure 7

ALI promotes EnAo physiologically-relevant gene expression programs, relative to Figure 7.

a. Cells in SC-EnAos were annotated into three cell types according to the cannonical EnSC markers (*DCN* and *LUM)*, EnEC markers (*EPCAM* and *KRT18)* and ciliated cells markers (*FOXJ1* and *DYNLRB2*) in SC-EnAo.

b. Representative GO terms of upregulated genes in ALI-EnSCs, compared to SC-EnSCs.

c. UMAP plots of decidualization genes PRL and IGFBP1 in EnSCs of ALI-EnAos and SC-EnAos (The percentage represents the proportion of positively expressed cells).

d. Volcano plots showing DEGs in unciliated epithelium between ALI-EnAos and SC-EnAos.

e. Representative GO terms of the DEGs in unciliated cells in ALI-EnAos, compared to SC-EnAos.

f. Heat maps showing distinct expression dynamics of representative genes related to endometrial receptivity array (ERA) under E_2 and E_2+P4+cAMP treatment to mimic proliferative and secretory phases in ALI-EnAos (left) and SC-EnAos (right). Gene expression levels were normalized.

References

[1]a) C. A. Finn, *Q Rev Biol* **1998**, *73* (2), 163, https://doi.org/10.1086/420183; b) J. Evans, L. A. Salamonsen, A. Winship, E. Menkhorst, G. Nie, C. E. Gargett, E. Dimitriadis, *Nat Rev Endocrinol* **2016**, *12* (11), 654, https://doi.org/10.1038/nrendo.2016.116.

[2] a) E. R. Norwitz, D. J. Schust, S. J. Fisher, *N Engl J Med* **2001**, *345* (19), 1400, https://doi.org/10.1056/NEJMra000763; b) B. C. Paria, J. Reese, S. K. Das, S. K. Dey, *Science* **2002**, *296* (5576), 2185, https://doi.org/10.1126/science.1071601.

[3] a) A. Psychoyos, *Ann N Y Acad Sci* **1986**, *476*, 36, https://doi.org/10.1111/j.1749-6632.1986.tb20920.x; b) W.-g. Ma, H. Song, S. K. Das, B. C. Paria, S. K. Dey, *Proceedings of the National Academy of Sciences* **2003**, *100* (5), 2963, https://doi.org/doi:10.1073/pnas.0530162100.

[4] a) J. D. Aplin, P. T. Ruane, *J Cell Sci* **2017**, *130* (1), 15, https://doi.org/10.1242/jcs.175943; b) J. L. James, A. M. Carter, L. W. Chamley, *Placenta* **2012**, *33* (5), 327, https://doi.org/10.1016/j.placenta.2012.01.020; c) B.

Gellersen, J. J. Brosens, *Endocr Rev* **2014**, *35* (6), 851, https://doi.org/10.1210/er.2014-1045.
[5] a) M. A. Lancaster, J. A. Knoblich, *Science* **2014**, *345* (6194), 1247125, https://doi.org/10.1126/science.1247125; b) T. Nakano, S. Ando, N. Takata, M. Kawada, K. Muguruma, K. Sekiguchi, K. Saito, S. Yonemura, M. Eiraku, Y. Sasai, *Cell Stem Cell* **2012**, *10* (6), 771, https://doi.org/10.1016/j.stem.2012.05.009; c) J. R. Spence, C. N. Mayhew, S. A. Rankin, M. F. Kuhar, J. E. Vallance, K. Tolle, E. E. Hoskins, V. V. Kalinichenko, S. I. Wells, A. M. Zorn, N. F. Shroyer, J. M. Wells, *Nature* **2011**, *470* (7332), 105, https://doi.org/10.1038/nature09691.
[6] a) M. Y. Turco, L. Gardner, J. Hughes, T. Cindrova-Davies, M. J. Gomez, L. Farrell, M. Hollinshead, S. G. E. Marsh, J. J. Brosens, H. O. Critchley, B. D. Simons, M. Hemberger, B. K. Koo, A. Moffett, G. J. Burton, *Nat Cell Biol* **2017**, *19* (5), 568, https://doi.org/10.1038/ncb3516; b) M. Boretto, B. Cox, M. Noben, N. Hendriks, A. Fassbender, H. Roose, F. Amant, D. Timmerman, C. Tomassetti, A. Vanhie, C. Meuleman, M. Ferrante, H. Vankelecom, *Development* **2017**, *144* (10), 1775, https://doi.org/10.1242/dev.148478.
[7] a) A. Kobayashi, R. R. Behringer, *Nat Rev Genet* **2003**, *4* (12), 969, https://doi.org/10.1038/nrg1225; b) A. Roy, M. M. Matzuk, *Nat Rev Endocrinol* **2011**, *7* (9), 517, https://doi.org/10.1038/nrendo.2011.79.
[8] H. Okada, T. Tsuzuki, H. Murata, *Reprod Med Biol* **2018**, *17* (3), 220, https://doi.org/10.1002/rmb2.12088.
[9] a) J. J. Brosens, M. S. Salker, G. Teklenburg, J. Nautiyal, S. Salter, E. S. Lucas, J. H. Steel, M. Christian, Y. W. Chan, C. M. Boomsma, J. D. Moore, G. M. Hartshorne, S. Sucurovic, B. Mulac-Jericevic, C. J. Heijnen, S. Quenby, M. J. Koerkamp, F. C. Holstege, A. Shmygol, N. S. Macklon, *Sci Rep* **2014**, *4*, 3894, https://doi.org/10.1038/srep03894; b) M. Salker, G. Teklenburg, M. Molokhia, S. Lavery, G. Trew, T. Aojanepong, H. J. Mardon, A. U. Lokugamage, R. Rai, C. Landles, B. A. Roelen, S. Quenby, E. W. Kuijk, A. Kavelaars, C. J. Heijnen, L. Regan, N. S. Macklon, J. J. Brosens, *PLoS One* **2010**, *5* (4), e10287, https://doi.org/10.1371/journal.pone.0010287.
[10] a) T. M. Rawlings, K. Makwana, D. M. Taylor, M. A. Mole, K. J. Fishwick, M. Tryfonos, J. Odendaal, A. Hawkes, M. Zernicka-Goetz, G. M. Hartshorne, J. J. Brosens, E. S. Lucas, *Elife* **2021**, *10*, https://doi.org/10.7554/eLife.69603; b) V. C. Cheung, C. Y. Peng, M. Marinic, N. J. Sakabe, I. Aneas, V. J. Lynch, C. Ober, M. A. Nobrega, J. A. Kessler, *Cell Rep* **2021**, *35* (7), 109138, https://doi.org/10.1016/j.celrep.2021.109138; c) T. Wiwatpanit, A. R. Murphy, Z. Lu, M. Urbanek, J. E. Burdette, T. K. Woodruff, J. J. Kim, *J Clin Endocrinol Metab* **2020**, *105* (3), https://doi.org/10.1210/clinem/dgz100; d) A. R. Murphy, T. Wiwatpanit, Z. Lu, B. Davaadelger, J. J. Kim, *J Vis Exp* **2019**, (152), https://doi.org/10.3791/60384; e) Y. Abbas, L. G. Brunel, M. S. Hollinshead, R. C. Fernando, L. Gardner, I. Duncan, A. Moffett, S. Best, M. Y. Turco, G. J. Burton, R. E. Cameron, *Interface Focus* **2020**, *10* (2), 20190079, https://doi.org/10.1098/rsfs.2019.0079.
[11] a) M. P. Snijders, A. F. de Goeij, M. J. Debets-Te Baerts, M. J. Rousch, J. Koudstaal, F. T. Bosman, *J Reprod Fertil* **1992**, *94* (2), 363,

https://doi.org/10.1530/jrf.0.0940363; b) B. A. Lessey, A. P. Killam, D. A. Metzger, A. F. Haney, G. L. Greene, K. S. McCarty, Jr., *J Clin Endocrinol Metab* **1988**, *67* (2), 334, https://doi.org/10.1210/jcem-67-2-334.
[12]a) L. Craciunas, I. Gallos, J. Chu, T. Bourne, S. Quenby, J. J. Brosens, A. Coomarasamy, *Hum Reprod Update* **2019**, *25* (2), 202, https://doi.org/10.1093/humupd/dmy044; b) P. Diaz-Gimeno, J. A. Horcajadas, J. A. Martinez-Conejero, F. J. Esteban, P. Alama, A. Pellicer, C. Simon, *Fertil Steril* **2011**, *95* (1), 50, https://doi.org/10.1016/j.fertnstert.2010.04.063.
[13]a) X. Ye, *Trends Endocrinol Metab* **2020**, *31* (2), 165, https://doi.org/10.1016/j.tem.2019.11.008; b) R. Demir, U. A. Kayisli, C. Celik-Ozenci, E. T. Korgun, A. Y. Demir-Weusten, A. Arici, *Placenta* **2002**, *23* (8-9), 672, https://doi.org/10.1053/plac.2002.0841.
[14]a) M. Prunieras, M. Regnier, D. Woodley, *J Invest Dermatol* **1983**, *81* (1 Suppl), 28s, https://doi.org/10.1111/1523-1747.ep12540324; b) M. J. Whitcutt, K. B. Adler, R. Wu, *In Vitro Cell Dev Biol* **1988**, *24* (5), 420, https://doi.org/10.1007/BF02628493.
[15]a) A. A. Pezzulo, T. D. Starner, T. E. Scheetz, G. L. Traver, A. E. Tilley, B. G. Harvey, R. G. Crystal, P. B. McCray, Jr., J. Zabner, *Am J Physiol Lung Cell Mol Physiol* **2011**, *300* (1), L25, https://doi.org/10.1152/ajplung.00256.2010; b) D. Cozens, E. Sutherland, F. Marchesi, G. Taylor, C. C. Berry, R. L. Davies, *Sci Rep* **2018**, *8* (1), 14893, https://doi.org/10.1038/s41598-018-33180-w.
[16]S. Chen, J. Schoen, *Reprod Domest Anim* **2019**, *54 Suppl 3*, 38, https://doi.org/10.1111/rda.13481.
[17]a) S. Chen, S. E. Palma-Vera, M. Langhammer, S. P. Galuska, B. C. Braun, E. Krause, A. Lucas-Hahn, J. Schoen, *Sci Rep* **2017**, *7*, 42298, https://doi.org/10.1038/srep42298; b) S. Chen, J. Schoen, *Methods Mol Biol* **2021**, *2273*, 251, https://doi.org/10.1007/978-1-0716-1246-0_18.
[18]S. Haider, M. Gamperl, T. R. Burkard, V. Kunihs, U. Kaindl, S. Junttila, C. Fiala, K. Schmidt, S. Mendjan, M. Knofler, P. A. Latos, *Endocrinology* **2019**, *160* (10), 2282, https://doi.org/10.1210/en.2019-00314.
[19]W. Wang, F. Vilella, P. Alama, I. Moreno, M. Mignardi, A. Isakova, W. Pan, C. Simon, S. R. Quake, *Nat Med* **2020**, *26* (10), 1644, https://doi.org/10.1038/s41591-020-1040-z.
[20]J. S. Gnecco, A. Brown, K. Buttrey, C. Ives, B. A. Goods, L. Baugh, V. Hernandez-Gordillo, M. Loring, K. Isaacson, L. G. Griffith, *SSRN: https://ssrn.com/abstract=4160140 or http://dx.doi.org/10.2139/ssrn.4160140* **2022**, https://doi.org/10.1101/2021.09.30.462577.
[21]H. C. Fitzgerald, P. Dhakal, S. K. Behura, D. J. Schust, T. E. Spencer, *Proceedings of the National Academy of Sciences* **2019**, *116* (46), 23132, https://doi.org/10.1073/pnas.1915389116.
[22]a) S. P. Choksi, G. Lauter, P. Swoboda, S. Roy, *Development* **2014**, *141* (7), 1427, https://doi.org/10.1242/dev.074666; b) A. Meunier, J. Azimzadeh, *Cold Spring Harb Perspect Biol* **2016**, *8* (12), https://doi.org/10.1101/cshperspect.a028233; c) R. MASTERTON, E. M. ARMSTRONG, I. A. R. MORE, *Reproduction* **1975**, *42* (3), 537, https://doi.org/10.1530/jrf.0.0420537; d) I. A. R. More, R. G. Masterton,

Reproduction **1976**, *47* (1), 19, https://doi.org/10.1530/jrf.0.0470019.
[23]N. Spassky, A. Meunier, *Nat Rev Mol Cell Biol* **2017**, *18* (7), 423, https://doi.org/10.1038/nrm.2017.21.
[24]a) C. Bartosch, J. M. Lopes, J. Beires, M. Sousa, *Reprod Sci* **2011**, *18* (6), 525, https://doi.org/10.1177/1933719110392055; b) A. H. Fruin, J. R. Tighe, *J Obstet Gynaecol Br Commonw* **1967**, *74* (1), 93, https://doi.org/10.1111/j.1471-0528.1967.tb03941.x; c) N. N. Amso, J. Crow, J. Lewin, R. W. Shaw, *Hum Reprod* **1994**, *9* (12), 2234, https://doi.org/10.1093/oxfordjournals.humrep.a138429.
[25]L. Garcia-Alonso, L. F. Handfield, K. Roberts, K. Nikolakopoulou, R. C. Fernando, L. Gardner, B. Woodhams, A. Arutyunyan, K. Polanski, R. Hoo, C. Sancho-Serra, T. Li, K. Kwakwa, E. Tuck, V. Lorenzi, H. Massalha, M. Prete, V. Kleshchevnikov, A. Tarkowska, T. Porter, C. I. Mazzeo, S. van Dongen, M. Dabrowska, V. Vaskivskyi, K. T. Mahbubani, J. E. Park, M. Jimenez-Linan, L. Campos, V. Y. Kiselev, C. Lindskog, P. Ayuk, E. Prigmore, M. R. Stratton, K. Saeb-Parsy, A. Moffett, L. Moore, O. A. Bayraktar, S. A. Teichmann, M. Y. Turco, R. Vento-Tormo, *Nat Genet* **2021**, *53* (12), 1698, https://doi.org/10.1038/s41588-021-00972-2.
[26]V. K. Maurya, F. J. DeMayo, J. P. Lydon, *Front Cell Dev Biol* **2021**, *9*, 640907, https://doi.org/10.3389/fcell.2021.640907.
[27]J. Y. Park, S. Mani, G. Clair, H. M. Olson, V. L. Paurus, C. K. Ansong, C. Blundell, R. Young, J. Kanter, S. Gordon, A. Y. Yi, M. Mainigi, D. D. Huh, *Nat Commun* **2022**, *13* (1), 1252, https://doi.org/10.1038/s41467-022-28663-4.
[28]a) S. D. Smith, R. H. Choudhury, P. Matos, J. A. Horn, S. J. Lye, C. E. Dunk, J. D. Aplin, R. L. Jones, L. K. Harris, *Histol Histopathol* **2016**, *31* (5), 557, https://doi.org/10.14670/HH-11-696; b) Y. Abbas, A. Carnicer-Lombarte, L. Gardner, J. Thomas, J. J. Brosens, A. Moffett, A. M. Sharkey, K. Franze, G. J. Burton, M. L. Oyen, *Hum Reprod* **2019**, *34* (10), 1999, https://doi.org/10.1093/humrep/dez139.
[29]A. Glentis, V. Gurchenkov, D. Matic Vignjevic, *Cell Adh Migr* **2014**, *8* (3), 236, https://doi.org/10.4161/cam.28733.
[30]a) D. R. Cochrane, K. R. Campbell, K. Greening, G. C. Ho, J. Hopkins, M. Bui, J. M. Douglas, V. Sharlandjieva, A. D. Munzur, D. Lai, M. DeGrood, E. W. Gibbard, S. Leung, N. Boyd, A. S. Cheng, C. Chow, J. L. Lim, D. A. Farnell, S. Kommoss, F. Kommoss, A. Roth, L. Hoang, J. N. McAlpine, S. P. Shah, D. G. Huntsman, *J Pathol* **2020**, *252* (2), 201, https://doi.org/10.1002/path.5511; b) M. Marinic, S. Rana, V. J. Lynch, *Placenta* **2020**, *101*, 75, https://doi.org/10.1016/j.placenta.2020.08.017.
[31]Z. Gu, J. Guo, J. Zhai, G. Feng, X. Wang, Z. Gao, K. Li, S. Ji, L. Wang, Y. Xu, X. Chen, Y. Wang, S. Guo, M. Yang, L. Li, H. Han, L. Jiang, Y. Wen, L. Wang, J. Hao, W. Li, S. Wang, H. Wang, Q. Gu, *Adv Sci (Weinh)* **2022**, e2202282, https://doi.org/10.1002/advs.202202282.
[32]Z. Richards, T. McCray, J. Marsili, M. L. Zenner, J. T. Manlucu, J. Garcia, A. Kajdacsy-Balla, M. Murray, C. Voisine, A. B. Murphy, S. A. Abdulkadir, G. S. Prins, L. Nonn, *iScience* **2019**, *12*, 304, https://doi.org/10.1016/j.isci.2019.01.028.
[33]S. Bergmann, M. Schindler, C. Munger, C. A. Penfold, T. E. Boroviak, *Commun Biol* **2021**, *4* (1), 749, https://doi.org/10.1038/s42003-021-02233-8.
[34]F. W. Bazer, T. E. Spencer, G. A. Johnson, R. C. Burghardt, G. Wu,

Reproduction **2009**, *138* (2), 195, https://doi.org/10.1530/REP-09-0158.
[35]a) S. Queckborner, E. Syk Lundberg, K. Gemzell-Danielsson, L. C. Davies, *Stem Cell Res Ther* **2020**, *11* (1), 15, https://doi.org/10.1186/s13287-019-1496-2; b) H. Murata, S. Tanaka, H. Okada, *J Clin Med* **2021**, *10* (2), https://doi.org/10.3390/jcm10020351.
[36]a) Y. Chen, J. Li, J. K. Xiao, L. Xiao, B. W. Xu, C. Li, *Cancer Cell Int* **2021**, *21* (1), 90, https://doi.org/10.1186/s12935-021-01780-8; b) Y. Bian, G. Gao, Q. Zhang, H. Qian, L. Yu, N. Yao, J. Qian, B. Liu, X. Qian, *Cancer Biol Ther* **2019**, *20* (6), 886, https://doi.org/10.1080/15384047.2019.1579959.
[37]a) D. Munoz-Espin, M. Serrano, *Nat Rev Mol Cell Biol* **2014**, *15* (7), 482, https://doi.org/10.1038/nrm3823; b) J. M. van Deursen, *Nature* **2014**, *509* (7501), 439, https://doi.org/10.1038/nature13193; c) E. S. Lucas, P. Vrljicak, J. Muter, M. M. Diniz-da-Costa, P. J. Brighton, C. S. Kong, J. Lipecki, K. J. Fishwick, J. Odendaal, L. J. Ewington, S. Quenby, S. Ott, J. J. Brosens, *Commun Biol* **2020**, *3* (1), 37, https://doi.org/10.1038/s42003-020-0763-1.
[38]K. M. Kim, J. H. Noh, M. Bodogai, J. L. Martindale, X. Yang, F. E. Indig, S. K. Basu, K. Ohnuma, C. Morimoto, P. F. Johnson, A. Biragyn, K. Abdelmohsen, M. Gorospe, *Genes Dev* **2017**, *31* (15), 1529, https://doi.org/10.1101/gad.302570.117.
[39]X. Fan, S. Krieg, J. Y. Hwang, S. Dhal, C. J. Kuo, B. L. Lasley, R. M. Brenner, N. R. Nayak, *Endocrinology* **2012**, *153* (3), 1063, https://doi.org/10.1210/en.2011-1826.
[40]S. Tulac, N. R. Nayak, L. C. Kao, M. Van Waes, J. Huang, S. Lobo, A. Germeyer, B. A. Lessey, R. N. Taylor, E. Suchanek, L. C. Giudice, *J Clin Endocrinol Metab* **2003**, *88* (8), 3860, https://doi.org/10.1210/jc.2003-030494.
[41]V. D. Venkata, M. F. B. Jamaluddin, J. Goad, H. R. Drury, M. A. Tadros, R. Lim, A. Karakoti, R. O'Sullivan, Y. Ius, K. Jaaback, P. Nahar, P. S. Tanwar, *Proc Natl Acad Sci U S A* **2022**, *119* (30), e2118054119, https://doi.org/10.1073/pnas.2118054119.
[42]B. A. Lessey, A. O. Ilesanmi, M. A. Lessey, M. Riben, J. E. Harris, K. Chwalisz, *Am J Reprod Immunol* **1996**, *35* (3), 195, https://doi.org/10.1111/j.1600-0897.1996.tb00031.x.
[43]a) Q. Li, A. Kannan, F. J. DeMayo, J. P. Lydon, P. S. Cooke, H. Yamagishi, D. Srivastava, M. K. Bagchi, I. C. Bagchi, *Science* **2011**, *331* (6019), 912, https://doi.org/10.1126/science.1197454; b) H. Cho, H. Okada, T. Tsuzuki, A. Nishigaki, K. Yasuda, H. Kanzaki, *Fertil Steril* **2013**, *99* (1), 248, https://doi.org/10.1016/j.fertnstert.2012.08.056; c) M. Christian, X. Zhang, T. Schneider-Merck, T. G. Unterman, B. Gellersen, J. O. White, J. J. Brosens, *J Biol Chem* **2002**, *277* (23), 20825, https://doi.org/10.1074/jbc.M201018200.
[44]I. Mukherjee, S. Roy, S. Chakrabarti, *Front Genet* **2019**, *10,* 23, https://doi.org/10.3389/fgene.2019.00023.
[45]a) P. Carmeliet, M. Baes, *N Engl J Med* **2008**, *358* (23), 2511, https://doi.org/10.1056/NEJMcibr0802500; b) J. Sun, H. Shen, L. Shao, X. Teng, Y. Chen, X. Liu, Z. Yang, Z. Shen, *Stem Cell Res Ther* **2020**, *11* (1), 373, https://doi.org/10.1186/s13287-020-01881-7.
[46]F. Mauri, C. Schepkens, G. Lapouge, B. Drogat, Y. Song, I. Pastushenko, S.

Rorive, J. Blondeau, S. Golstein, Y. Bareche, M. Miglianico, E. Nkusi, M. Rozzi, V. Moers, A. Brisebarre, M. Raphael, C. Dubois, J. Allard, B. Durdu, F. Ribeiro, C. Sotiriou, I. Salmon, J. Vakili, C. Blanpain, *Nat Cancer* **2021**, *2* (11), 1152, https://doi.org/10.1038/s43018-021-00287-5.

[47]a) H. Heidari-Khoei, F. Esfandiari, A. Moini, S. Yari, M. Saber, M. Ghaffari Novin, A. Piryaei, H. Baharvand, *Exp Cell Res* **2022**, *417* (1), 113205, https://doi.org/10.1016/j.yexcr.2022.113205; b) T. Cindrova-Davies, X. Zhao, K. Elder, C. J. P. Jones, A. Moffett, G. J. Burton, M. Y. Turco, *Commun Biol* **2021**, *4* (1), 651, https://doi.org/10.1038/s42003-021-02194-y.

[48]a) H. Takeda, S. Kataoka, M. Nakayama, M. A. E. Ali, H. Oshima, D. Yamamoto, J. W. Park, Y. Takegami, T. An, N. A. Jenkins, N. G. Copeland, M. Oshima, *Proc Natl Acad Sci U S A* **2019**, *116* (31), 15635, https://doi.org/10.1073/pnas.1904714116; b) G. Schwank, A. Andersson-Rolf, B. K. Koo, N. Sasaki, H. Clevers, *PLoS One* **2013**, *8* (10), e76871, https://doi.org/10.1371/journal.pone.0076871; c) G. Schwank, B. K. Koo, V. Sasselli, J. F. Dekkers, I. Heo, T. Demircan, N. Sasaki, S. Boymans, E. Cuppen, C. K. van der Ent, E. E. Nieuwenhuis, J. M. Beekman, H. Clevers, *Cell Stem Cell* **2013**, *13* (6), 653, https://doi.org/10.1016/j.stem.2013.11.002.
